# A biological control model to manage the vector and the infection of *Xylella fastidiosa* on olive trees

**DOI:** 10.1371/journal.pone.0232363

**Published:** 2020-04-30

**Authors:** Antonella Liccardo, Annalisa Fierro, Francesca Garganese, Ugo Picciotti, Francesco Porcelli

**Affiliations:** 1 Physics Department, Università degli Studi di Napoli “Federico II”, Napoli, Italy; 2 Consiglio Nazionale delle Ricerche (CNR)—Institute Superconductors, oxides and other innovative materials and devices (SPIN), Napoli, Italy; 3 Dipartimento di Scienze del Suolo, della Pianta e degli Alimenti, University of Bari Aldo Moro, Apulia, Italy; 4 Departamento de Ciencias del Mar y Biologia Aplicada, Universidad de Alicante, Alicante, Spain; 5 CIHEAM IAMB, Valenzano, Italy; Universita del Salento, ITALY

## Abstract

*Xylella fastidiosa pauca* ST53 is the bacterium responsible for the Olive Quick Decline Syndrome that has killed millions of olive trees in Southern Italy. A recent work demonstrates that a rational integration of vector and transmission control measures, into a strategy based on chemical and physical control means, can manage *Xylella fastidiosa* invasion and impact below an acceptable economic threshold. In the present study, we propose a biological alternative to the chemical control action, which involves the predetermined use of an available natural enemy of *Philaenus spumarius*, i.e., *Zelus renardii*, for adult vector population and infection biocontrol. The paper combines two different approaches: a laboratory experiment to test the predation dynamics of *Zelus renardii* on *Philaenus spumarius* and its attitude as candidate for an inundation strategy; a simulated experiment of inundation, to preliminary test the efficacy of such strategy, before eventually proceeding to an in-field experimentation. With this double-fold approach we show that an inundation strategy with *Zelus renardii* has the potential to furnish an efficient and “green” solution to *Xylella fastidiosa* invasion, with a reduction of the pathogen incidence below 10%. The biocontrol model presented here could be promising for containing the impact and spread of Xylella fastidiosa, after an in-field validation of the inundation technique. Saving the fruit orchard, the production and the industry in susceptible areas could thus become an attainable goal, within comfortable parameters for sustainability, environmental safety, and effective plant health protection in organic orchard management.

## Introduction

*Xylella fastidiosa* (Wells et al., 1987) *pauca* ST53 (Xf) is the bacterium responsible for the Olive Quick Decline Syndrome (OQDS), a devastating plant disease that has killed millions of olive trees in Southern Italy [1], [2], [3], [4], [5]. The main xylem-sap feeder vector of Xf is the adult Meadow Spittlebug, *Philaenus spumarius* (Ps) (Linnaeus, 1758) (L.) (Hemiptera Aphrophoridae) [6], which acquires the bacterium while feeding on infected plants, and transmits it. Vector control is expected to be the main action to manage insect-borne pathogens and to contain the disease. In [7] authors propose an epidemiological lattice model for the pathogen invasion of olive orchard aimed at identifying an appropriate strategy for arresting the infection, built on vector management throughout the entire vector’s life cycle. In particular, the olive orchard is represented as a simple square lattice with olive trees and herbaceous vegetation distributed on the lattice sites in a realistic way; adult vectors are particles moving on the lattice according to rules dictated by the interplay between vector and vegetation life cycles and by phenology; the tree’s epidemic process is modelled as a stochastic SIR (Susceptible, Infected, Removed) model [8] on a lattice [9, 10]. An Integrated Pest Management strategy, based on tailoring, timing, and tuning of available control actions, is superimposed on this baseline model, enabling authors to demonstrate that it is possible to stop the Xf invasion in a two year interval, by a rational and quantitative vector and infection control strategy. The pest management is based on the integration of diverse chemical and physical control means versus different steps of vector’s life cycle. In the proposed IPM strategy, egg and juvenile stages are managed by mechanical control actions, while some superimposed preventive and protective chemical control actions manage the adults. Chemical control, acting on the Xf vectors, indirectly impedes or minimize the infection thus preventing Xf to invade new territories.

**Fig 1:**
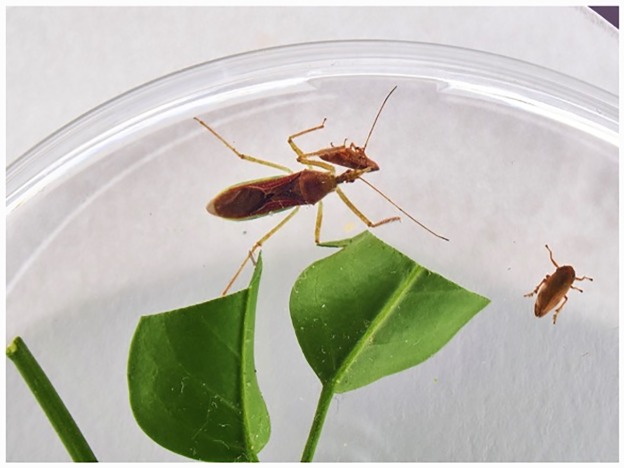
Zr preying an adult Ps.

In the present study we propose a biological control action alternative to the chemical one described in [1], which involves the predetermined use of a natural enemy of Ps i.e. *Zelus renardii* (Zr) [11, 12] (Hemiptera Reduviidae), shown in Fig 1, against the adult vectors.

When attempting to manage a pest, the knowledge of the vector responsible for the transmission of a pathogen, and the subsequent identification of one or a guild of its natural enemies, open the way for biological control actions, possibly avoiding the side-effects of synthetic chemical insecticides. Indeed, biological control actions do not produce chemical residues, wishing to be pest-specific and selective enough. Therefore, biological control agents are more compatible with sustainable plant protection [13].

Different approaches are reported to the pest biocontrol [13] based on the usage of natural enemies of the target species. The importation or classical strategies are useful in cases of alien introduced noxious organisms, i.e. when a pest appears in a new area in which pathogen/natural enemies, from the origin area of the pest, are absent ([14–17]). The augmentative strategies (i.e. inoculation and inundation), instead, are useful in cases in which natural enemies are already present in the area but their number or life cycle do not fit with the aim of eradicating or containing the pest. The pest control may be achieved by released individuals and their progeny, as in the inoculation [18], or directly by the released biological control agents, that should be able to kill a huge number of pest vectors, without relying on their progeny, as in the inundation strategy [19]. This inundation strategy is particularly recommended if the target organism action is very fast, and a rapid control is necessary in order to avoid destructive effects on the recipient and losing individuals. Finally with the enhancement or conservation strategy, feeding and environmental strategies are adopted with the aim of enhancing the natural enemy population, and consequently its efficiency in mitigating the pest, [20].

In the case of OQSD, the Xf bacterium is believed to be an exotic pathogen introduced in Europe by the trade of ornamental asymptomatic coffee plants from Costa Rica [21]. However, its vector (Ps) is an indigenous insect, well established in Italy and in all EU countries.

The fact that Ps was not previously associated with Xf in Europe, has meant that the scrutiny for the natural enemy guild of European Ps and other xylem-feeders possible Xf vectors, started quite recently. The actual knowledge [22], [23], [24] shows the global and Italian regional poorness of the antagonists of Xf vectors. Literature [25], [26], [27] reports as antagonists: *Ooctonus americanus* (Girault, 1916), *Ooctonus* sp. (Mymaridae), *Tumidiscapus* sp., *Centrodora* sp. (Eulophidae) all Hymenoptera; *Agamermis decaudata* (Cobb, Steiner, and Christie, 1923) (Nematoda Mermithidae) and *Entomophthora* sp. (Fungi). The EFSA External Scientific Report [22] asserts that the palearctic origin of Ps and other Aphrophoridae vectors of Xf ST53 makes not feasible the inoculative biological control and that, apart for some generalist predators, very few information, if any, on other natural enemies is available. The present knowledge makes it difficult or impossible to imagine a biocontrol action based on a conservation approach, as well as for spiders that act as a guild of natural biocontrol agent [28].

In the present study, we propose to adopt a natural enemy of Ps, Zr, to carry out inundative biocontrol actions against the vectors of Xf ST53.

Zr is commonly known as leafhopper assassin bug [29]–[41] and the trivial name well describes its prey-preference. Kolenati first described the species Zr in [11] as pertaining to the genus *Zelus* Fabricius. Zr originates in California and the species was dedicated to Dr. Renard. The status of the genus *Zelus* was recently and comprehensively reviewed by [42].

Zr recently entered by human-mediated dispersion [43] many European and neighboring countries. It is present in Greece [37–39], Spain [12], [44], [45], Turkey [46], Albania [47] and Israel [48]. In Italy its presence was first reported in [49] and then declared established in [50], while [41] already considered Zr as established in Europe. The natural occurrence of the taxon for about ten or more years in many European and neighboring Mediterranean Countries considerably mitigates the concerns about its aggressivity to local biocoenosis.

Observations of Ables [36] also supported by [37] (and supplementary material available at: http://purl.fcla.edu/fcla/entomologist/browse) emphasize the well-known preference of Zr for disturbed environments. In particular, they are more abundant in urban environments, where they can find a plethora of available prey, less in agricultural ones and scarce in “wild” contexts. One can detect the predator occurrence basing on the egg batches, even after the egg hatching, because of the persistence of abandoned choria on leaves or different supports (see S1 Fig).

Our aim is to show that one or more inundative biocontrol actions have the potential to stop the spreading of Xf. The first inundation may be used either to replace the chemical control action already described in [1] or to counteract the survived vectors re-entering in olive orchard. In case of two inundations the first replaces the chemical control action(s) while the second targets the survived vectors re-entering in olive orchard during their pre-ovigerous and the ovigerous lifetime.

In this study we consider the impact of Zr on Xf vectors, not on other olive pests that are now secondary in comparison with the damage due to Ps.

As described in [13], the inundation is not given to create a new equilibrium but to act as a living insecticide, whose action is limited in space and time into closed or open olive orchards that are free or infected by *Xylella fastidiosa pauca* ST53.

The work consists in two distinct parts: 1) a feeding experiment aimed at evaluating the Zr attitude as Ps predator and at measuring the feeding time and rates; 2) a numerically simulated experiment aimed at evaluating the efficiency of a simulated inundation of an olive orchard with Zr, against the *Xylella fastidiosa* invasion.

## Materials and methods

### The feeding experiment

We conducted a laboratory experiment on a Zr population, by feeding the predator with living Ps as prey. The aim of the experiment was to measure the time spent to kill each vector, i.e. the sum of the attack time and the feeding time.

Actually, the *Zelus* population adopted for the experiment was a laboratory population that underwent a three-years-long continuous mass-breeding experience on living prey: *Drosophila melanogaster* (Meigen, 1830) wild strain, *Drosophila suzukii* (Matsumura, 1931) from fruit crop orchards, and *Megaselia scalaris* (Loew, 1866), collected in Bari County and reared in laboratory on a purposely made breeding ground. We note that the lab-reared *Zelus* individuals were selected by eliminating lineages that revealed lethal or unfavorable features. During the Zr breeding we considered thus unavoidable the occurrence of some degree of selection with respect to wild population.

#### Zr adults rearing

Experimented Zr were chosen from those available in the mass rearing established from 2015 [7] on living prey and each experiment engaged a different individual. To choose a Zr female for the arena, we firstly ensured that it preyed, after laying its last eggs batch. Adults Zr were reared isolated with living prey as specified below. Zr pairs were bi-weekly sorted for mating and placed in 9 cm diameter vented Petri dishes. After mating, females were re-isolated in Petri dishes, providing them prey and leaving them to lay egg batches (see S2 Fig). The batches were set apart and, 24 h after egg-hatching, young predators were isolated in a vented Petri or 125 cc flask to receive the same living prey-based diet, life-long. Wet cotton disks and/or filter paper pieces provided grip and a proper %RH in rearing boxes. We weekly transferred each Zr in a brand-new rearing box and replaced the equipment to reduce the contact of the predators with excrement, exuvia and other dirties, so improving the health status of the developing Zr. Daily inspections permitted further cleaning ad residues removal. In total we considered a bulk of 34 individuals of Zr bred with living prey. Each Zr received an individual ID code blindly reporting: date of hatching, date of moult/metamorphosis, type of diet, gender of the adult, date of death and apparent cause. All the experiments occurred in the “Entomologia Forense” accredited lab of DiSSPA—UNIBA Aldo Moro.

#### Mass rearing of living prey

Juveniles or pre-adults Zr were mass reared with living prey requiring a continuous supply of adult *D. melanogaster, D. suzukii*, or *M. scalaris*. The vinegar flies (VF) grew in four liter plexiglass flasks on meridic artificial substrate made up by 58.9 g of sucrose, 58.9 g of cornmeal, 2.5 g of agar, 50 g of brewer’s yeast, 0.5 g of methyl 4-hydroxybenzoate (methylparaben) and 1.7 ml of propionic acid in one liter of water. On alternate days, five flies were transferred from their rearing to Zr rearing boxes. The spotted wing *Drosophila* (SWD) were obtained and grew on ripen and overripen fruits available in the season put, indifferently, in one of the two halves of a petri plates placed in small breeding boxes. *Megaselia scalaris* (Scuttle flies: SF) adults were bred on exhausted wet espresso capsules into an opaque stopped wastebasket. We neither considered the nutritional composition and cost of the prey [51] nor the boundless *pabula* of the species [52] nor specialized diets [53]. SWD and SF breeding originate in nature and were occasionally integrated by wild individuals. VF, SWDs and SF were captured by small mouth aspirator to be transferred into the Zr breeding. 5th nymphs and adults Zr also fed on adult Muscidae or Calliphoridae bred in the laboratory, from maggot for fishing.

#### Emergency oligidic diet

An oligidic diet (Od) was occasionally offered to Zr in case of unavailability of living prey or during cold events in laboratory because of heating technical failures. Od is an artificial diet composed of raw organic material, chemically undefined. Our Od consisted of 200 g of fresh beef liver, 20 g of egg yolk and 30 ml of 30% sucrose solution in water. All was homogenised by a mixer. The diet was offered liquid by a micropipette in 0.25 ml aliquots per day or agar-jellified as small blocks. The Od was jellifies mixing 50 g of Od, 1 g of agar, and 1 g of ascorbic acid (antioxidant) in 100 ml of distilled water. The artificial diet was removed promptly as living prey were available again.

#### Rearing and experiment laboratory conditions

The lab conditions varied according to the seasons, with a minimum of 18°C and maximum of 25°C guaranteed by air conditioning in summer and heating in winter. Spring and fall temperatures were related to the outdoor ones. From April 1-st to May 14-th, the rearing temperature followed the external one; from May 15-th to October 14-th, temperatures were adjusted to 25°C; from October 15-th to November 14-th, the temperatures changed according to the outdoor ones; from November 15-th to March 31-st, the temperature was maintained to 18°C by heating, except on Saturdays and Sundays when the temperatures were related to the outdoor weather.

#### *Philaenus spumarius* adult’s collection

Ps adults used in experiments were collected in uncultivated, urban and peri-urban areas every day by sweeping herbs in dicot-rich areas and irrigated fields near Valenzano (BA—Italy) (see S3 Fig). Insects were collected by a Ps purposely-developed sweeping net, and moved into a 5 liters vented flask by a funnel secured on a small picnic table, purposely modified. The technique also collected parts of the plants hosting the vectors at the collection, giving some food for the vectors. The technique avoided the sudden depressurization normally due to mouth aspirators, also preventing to injure the vectors by a brutal management. We used for the experiments the fully active vectors only, i.e. those climbing on the flask walls. In the lab Ps were kept one more day singly in a flask with a Jasmine (*Jasminum officinale* L. Oleaceae) twig in water and exposed to Zr the day after, if fully active on the basis of excrements.

#### Experiment setting up, arena, feeding experiments, and data collection

**Fig 2:**
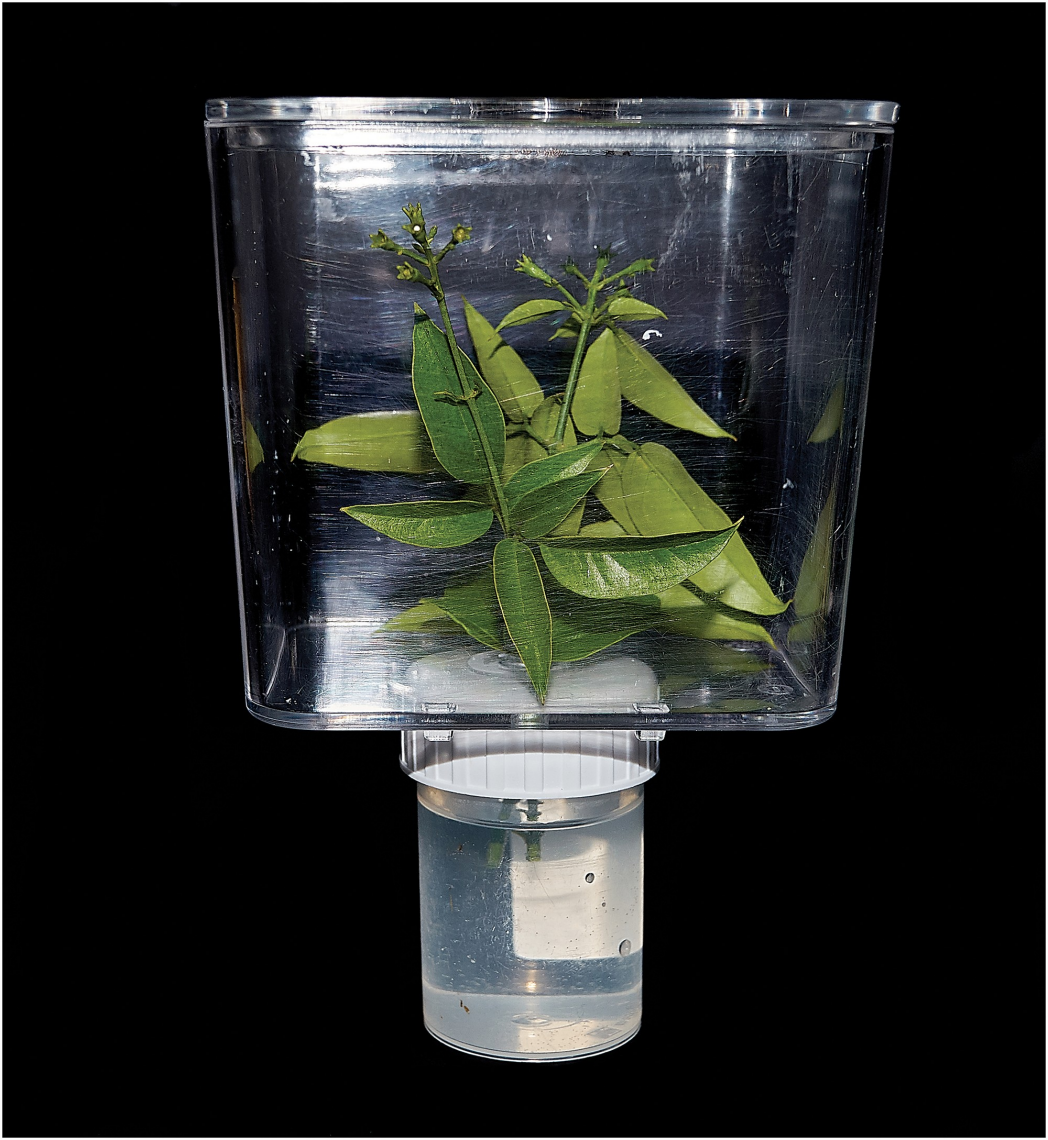
Arena for the experiments with a Ps adult inside.

To test the preying efficiency of Zr versus Ps, we used as arena a rectangular parallelepiped acrylic crystal-clear plastic box of 500 *cm*^3^ (10 × 10 × 5 *cm*^3^) as the arena (see Fig 2). The box was top-vented by a nylon mesh (0.8 × 0.2 *mm*″). The box lies on a water reserve to sustain a fresh Jasmine twig, used in the arena as a food source for Ps and to support the action of *Zelus*. Ps vitality in experimental conditions seemed not disturbed by artificial environment, in which they fed evidently on *Jasminum*, egested honeydew, and mated frequently. Predators were starved during the 24 hours preceding each day of trials and all tests were conducted in laboratory conditions described above. We used eight different predators (4 in 2016 and 4 in 2017) belonging to eight different rearing lineages started with the eggs of eight different females collected in eight localities near Bari, Valenzano (BA), Maglie (LE), and Foggia at months of distance each other. Predators were tested to prey on Ps adults, offering 5 prey at time in each replication (0.01 Ps/cm^3^) to observe the time of attack and feeding.

Observations run about 1 month, obtaining 40 replications in about 60 days, recording the time to attack and feeding time.

#### Predation parameters

In [54] authors suggested that reduviid predators act in a sequence of arousal, approach, capture, rostral probing, injection of toxic saliva, paralyzing, sucking, and post-predatory behaviour. Zr did not show all the steps clearly separated in our experience. We divide the steps into two phases, because the time in which Zr wanders until it grabs the prey is long enough to be measurable, while the capture, the rostral probing, the injection of toxic saliva and the paralyzing occur quickly and are not easy to be measured or even perceived. Thus we consider the two following steps

- step *I*: arousal, approach, capture, rostral probing, injection of toxic saliva and paralyzing the prey;
- step *II*: sucking the prey.

For step *I* we define the *time of attack* as the wandering time to the sudden fore-legs grabbing for prey catching, while for step *II* the *feeding time* lasts from the insertion of the stylets to the abandonment of the carcass. The action of the predator between the first and last vector predation is continuous and each predation time interval *τ*_tot_ originates from the sum of the two intervals.

### The model

The model describing the pathogen transmission by adult meadow spittlebugs was designed as in [1]. We briefly remind the structure.

The orchard was represented as a simple square lattice with a realistic distribution of olive trees and herbaceous vegetation, i.e. trees planted on parallel rows, at a distance *s*, with their canopies occupying the first and second nearest neighbour sites, and spontaneous herbs growing around the canopies. Each site of the lattice occupied by a tree represented a main branch of the tree, with a fixed number of twigs.

The introduction of Xf vectors in the orchard was timed on the forecasted appearance of adult spittlebugs (i.e. half of April, under typical weather conditions in South Italy). Indeed only adults can transmit the bacterium, the remaining part of the vector life cycle, embryonic and post-embryonic development, being not relevant for transmission because egg and juvenile stages lie on spontaneous herbs and are almost immotile. On the contrary, adults are able to abandon drying herbs, infesting the nearest growing-up plants, mainly olives [22].

**Fig 3:**
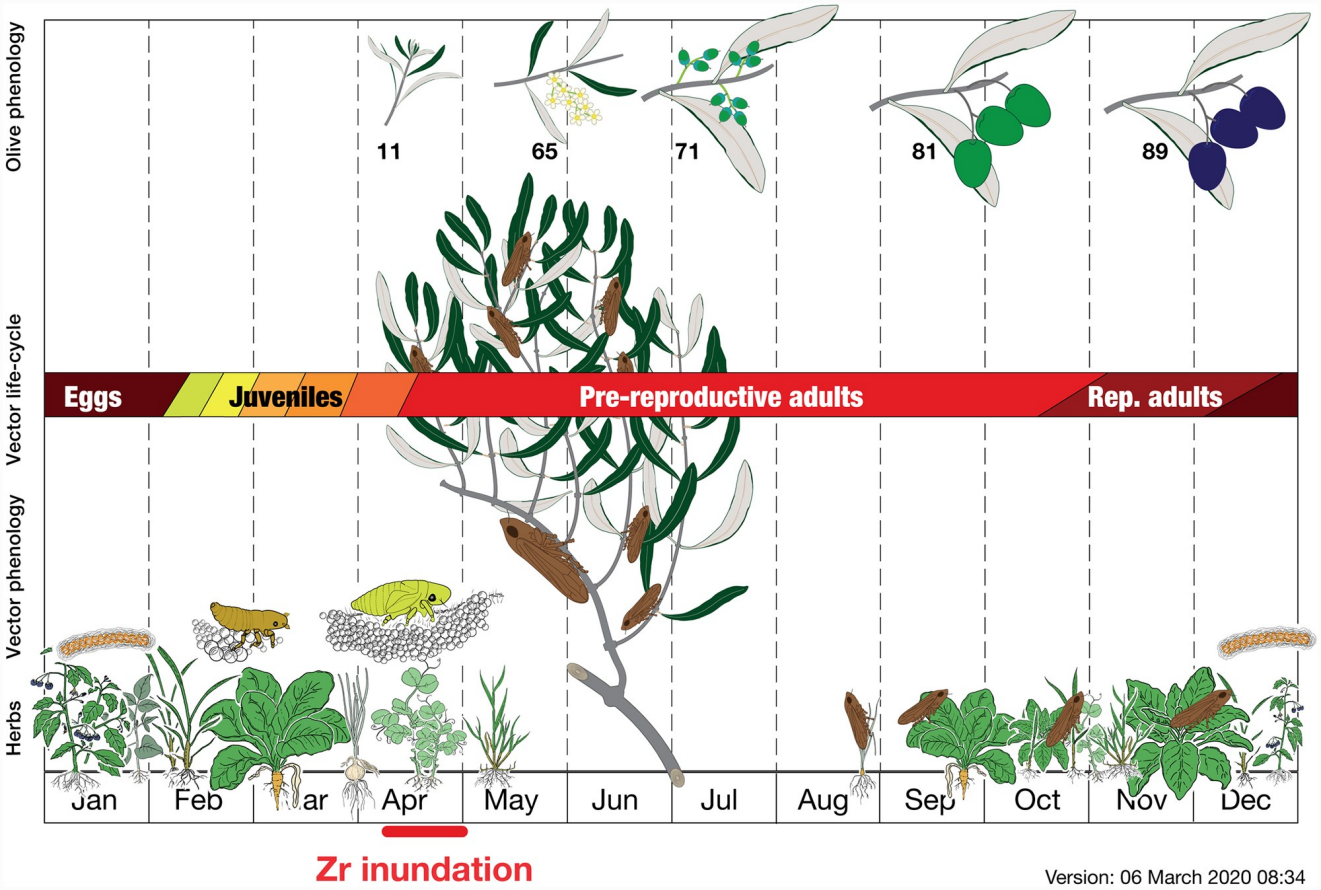
Scheme to show how the Zr inundation interplays with vectors and their host plants. From the bottom one can find the time in months, the herbs and the olive in the orchard with the vector life-cycle. On the image top five phenological milestone mark the orchard management and production. Numbers near the olive phenological drawing refers to the growth stages in [55].

The dynamic of adult insects consisted in the displacement on nearest neighbour sites and, within each site occupied by an olive branch, from twig to twig. We remind that adult Ps are xylem sap-feeder insects. However, as soon as olives harden (early summer), insects are not able to pierce anymore the olive wood and to suck the xylem, and thus they move on available vegetation to feed. The permanence of adult insects on olive trees is thus limited around the flowering period when olive twigs are tender (for the interplay of vector life cycle and vegetation phenology, see Fig 3).

In order to take in account different possible movements, as well as the previous seasonal feeding preferences, we introduced four different time-dependent probabilities corresponding to the movements from tree to tree, *p*_*tt*_, from herb to herb, *p*_*hh*_, from tree to herb, *p*_*th*_, and viceversa, *p*_*ht*_. Formally, vectors were treated as particles moving at random on the lattice with a preferential choice for sites occupied by trees in the olive flowering time (late spring—early summer) (*p*_*ht*_ > *p*_*th*_) and for herbaceous vegetation otherwise (*p*_*ht*_ < *p*_*th*_).

When feeding on infected trees, adults acquire the bacterium and transmit it, with no latency for the model, in other healthy or already infected trees during subsequent feedings. Once inside the tree, the bacterium multiplies in the xylem vessels, compromising the xylem sap circulation. As a consequence vectors avoid feeding on that xylem. The transmission period coincides with the adults mobility phase on olive trees. In order to represent this scenario, the transmission model on trees was structured as a SIR (Susceptible, Infected, Removed) model, with the susceptible (S) and the infected (I) being respectively the healthy and infected trees and the removed corresponding to symptomatic trees. In the present model the status “infected” was attributed to each tree having at least one infected twig. In our disease restraint strategy, we did not consider the possibility to cut down infected trees, since they did not take part anymore to the epidemic spreading (i.e. in the SIR terminology, the symptomatic trees are Removed).

The probability of adults to transmit Xf to olive trees was assumed to be constant, while the probability for an uninfected vector to acquire the bacterium was expressed as an appropriate function of the time occurred between the infection and the feeding act, to take in account that twigs have finite length and that the time required by Xf to propagate in the plant vessels is finite as well [1]. We assumed that egg and juvenile management occurred through mechanical control actions properly timed in winter and in April, respectively. The efficacy of such actions were cautiously assumed to be significantly below a realistic threshold. At the eclosion time, the number of adults appears strictly dependent on the efficacy of this preliminary management. The orchard was subsequently inundated with Zr preying over vectors.

Other experiments (see S4 Fig) show that Zr preys, with the same dynamic, other insects present on olive [56] that are neither vector of Xf, nor of other pests, like the *Issidae*. These insects are possible alternative prey for the Zr, and thus, for the sake of realism, they must be included in the simulation. Neglecting their presence would lead, indeed, to an overestimation of the feeding capacity of the predators on Ps and thus to an overestimatiation of the efficacy of the inundation strategy, that we want to avoid of course. In particular, *Latilica maculipes* (Melichar, 1906), collected and identified, on the basis of [57], lives in about 150 individuals per olive in such olive groves whose structure is compatible with the lattice model. For this reason we added in the model the presence of one alternative prey on olive trees. The Zr predation was shared between the two prey, following the frequencies of encounters, with a pure functional response to the available number of prey.

#### Model setup

The properties of the model were studied using a numerically simulated experiment. We performed 20 independent realisations of each experiment with different random generator seeds. The simulated data and their errors were evaluated respectively as mean values and standard deviations, over the independent processes. The code was developed in analogy with Monte Carlo simulations of physics models. The main difference is that here particles are replaced by insects, then the dynamics is not regulated by an Hamiltonian, but by biological constraints.

As in Ref. [1], each tree occupies 9 sites of the 2*d* lattice (the centre and all its nearest and second-nearest neighbours), each site corresponding to a tertiary branch of plants, with *N*_*twig*_ twigs per branch (*N*_*twig*_ is chosen equal to 12). Centres of two adjacent trees are put at distance *s*. Trees are characterised by an hardening time, *τ*_*hard*_ (tree becomes hard at *τ*_*hard*_ and becomes tender again in spring), extracted for each plant from a Gaussian distribution, once and for all. We chose the medium hardening time of twigs and the standard deviation equal to 185 days and 2 days, respectively. The health state of twigs and the time of infection were acquired every time step.

We assumed that the olive orchard was populated by a stable population of 1, 000, 000/ha Ps (as in [1]). Furthermore we assumed the presence of an equal number of alternative prey in the orchard, as preliminary working hypothesis. In the discussion session a sensitivity analysis, with different proportions with respect to the Ps population, is discussed. For the sake of simplicity, we assumed alternative prey to have similar mobility in the orchard and life cycle of the Ps population.

Insects were distributed at random on the lattice sites and introduced in time according to a Gaussian distribution centred on the mean eclosion time, *τ*_*ecl*_, put equal to 120 days, and with a standard deviation, *σ* = 5 days. In order to take into account that the eclosion does not start exactly the same day every year, the mean eclosion time is randomly chosen from *τ*_*ecl*_ − 3 to *τ*_*ecl*_ + 3 at the beginning of the year. The total number of insects, which appear after eclosion at new year, are evaluated as

$$\begin{array}{c} A^{y}=\frac{N_{P}^{y-1}}{2}\times \text{Off}\times \left(1-m\right)\times \left(1-{\text{eff}}_{e}\right)\times \left(1-{\text{eff}}_{j}\right) \end{array}$$ (1)

where $N_{P}^{y-1}$ is the number of adults surviving at the end of the year *y* − 1, Off is the number of eggs per female, *m* is the egg mortality, and eff_*e*_ and eff_*j*_ are the efficacy of egg and juvenile control actions, respectively. As in Ref. [1], we chose Off = 100, *m* = 98%, eff_*e*_ = eff_*j*_ = 70%. In winter all the survived insects die. The health state and the position of insects were acquired every time step.

As in Ref. [1], we considered two different scenarios: the closed case, where the orchard is isolated, and the open one, where the infection can propagate from nearest neighbour infected orchards. Thus, differently from the closed system, in the open one there were in-coming and out-going fluxes of insects through the boundary. The orchard was supposed to be a square with one of the boundaries adjacent to an infected orchard (Infected area), the opposite one adjacent to an uninfected one (Buffer area), and the other two boundaries adjacent to orchards that are homogeneous on the infection point of view, as in Fig 6 of Ref. [1]. We assumed a proportionality relation between the in-coming and the out-going flux of infected insects through a certain edge. In particular, the probability of the in-coming vectors to be infected were chosen in order to simulate an infection that spreads in one direction: through the southern edge the in-coming infected insects double the out-going ones, while through the northern edge the proportionality factor was chosen 1/10. Furthermore, we assumed a homogeneity of plant and vector healthy state on the other two edges, thus the proportionality factor on the East and West edges were fixed to 1.

In summary, the code developed in Ref. [1] was modified by the introduction of an alternative prey and by the inundation of the orchard by means of *N*_z_ predators, randomly distributed at forecasted vector eclosion time. In order to better understand how the releasing procedure influenced the evolution of the pest, we also considered the realistic case, in which the predators are released all in the same point, and the case, in which they are released in a short time window around *τ*_*ecl*_.

The core of simulation code was obviously given by insects (both prey and predator) dynamics. Schematically, the main routine called the other ones, respectively for

- memory allocation;
- definition of lattice structure;
- placement of olive trees and herbs on the lattice;
- regulation of birth, death and dynamics of insects, and, consequentially, spreading of the disease.

The routine, which regulates the dynamics, is called once daily.

**Predator dynamics**: We considered a daytime of 14 hours and 30 minutes in Apulia in May. In order to treat the worst case scenario, we subtracted a minimum time of 2h to the whole daylight time, to take in account time Zr dedicated to courtship and mating, egg laying and other biological activities, including escaping from non receptive partners, or other environmental disturbance. Thus we assumed that predators dedicate to hunting and feeding 12 hours and 30 minutes a day. This worst-case choice is supported by some experimental evidences: we observed that all the prey were killed long before the predation deadline. Furthermore, experiments conducted in 50cc Falcon tubes with one predator and plenty (15-20) of prey show that all the available prey were killed, but not necessarily consumed in 12h interval. Latest prey were just killed and discarded.

During the predation and feeding time, predator in site *i* catches a prey randomly chosen in the same site, if the time from the last feeding is larger than a value *τ*_f_. If there is no prey in site *i*, predator tries to move every 10 minutes until it finds a prey to eat. In details, for the *j*–th predator located at site *i*:

- A nearest neighbour destination site *f* is randomly chosen and individual moves to the destination site with probability 1;
- If some prey are present in the destination site, predator preys one of them, randomly chosen, with probability 1.
- Predator dies/abandons the orchard respectively in the closed/open scenario, if it does not eat for 5 days.

We repeat the above steps *N*_z_ times, where *N*_z_ is the current number of predators. The parameter *τ*_f_ is a best fit parameter fixed in order to guarantee that, for *N*_P_/*N*_z_ = 5, as in the experimental setup, on average, the time between one meal and the next is consistent with the time obtained by the feeding experiment, *τ*_tot_.

**Prey dynamics**: We assumed that prey try to move every half an hour, on the basis of published paper [58] and field observations and to permit a comparative discussion with [1].

In details, for the *j*–th prey located at site *i*:

1. If the site *i* is occupied by herb, a nearest neighbour destination site *f* is randomly chosen:
  - if *f* is occupied by herb, individual moves with probability *p*_*hh*_;
  - if *f* is occupied by a branch of an olive tree, individual moves with probability *p*_*ht*_.
2. If the site *i* is occupied by a branch of an olive tree, individual tries to move with equal probability to another twig or to the nearest neighbour destination site, *f*, randomly chosen on the lattice:
  - movements to twigs of the same branch are accepted with probability *p*_*tt*_;
  - movements to a different branch are accepted with probability *p*_*bb*_;
  - movements to herb are accepted with probability *p*_*th*_.
  The values of the probabilities, listed in Table 1, were fixed as in Ref [1], and depend on the specific period of the year and on the symptomatic status of infected tree.
  Movements to hard trees were always rejected. We assumed *p*_*bb*_ to be half of *p*_*tt*_. This choice was motivated by the morphological structure of branches and twigs.
3. After each movement, we control the state of health of plants and vectors:
  - healthy plants contract the infection from infected vectors with susceptibility *S*_*t*_;
  - healthy vectors acquire the bacterium with a transmission probability dependent on the time since infection, in order to take into account that twigs had finite length, but the infection process is point-like and bacteria propagation process into plant vessels requires a finite time. Assuming a bacteria propagation velocity of *v* = 5 cm/month and a twig length of roughly *L* = 15 cm, after 3 months from infection, the whole twig becomes infected and the susceptibility becomes equal to *S*_0_ (we choose *S*_0_ = 100%). Thus, we assumed the infection probability of a vector feeding at time *t* on a twig infected at time *t*_*inf*_ to be
    $$\begin{array}{c} S_{v}=\{\begin{array}{c} S_{0}\left(t-t_{inf}\right)\frac{v}{L}\;\left(t-t_{inf}\right)<3\;\text{months},\\ S_{0}\;\;\;\;\;\;\;\;\;\;\;\;\;\;\;\;\;\;\text{otherwise}. \end{array} \end{array}$$ (2)

**Table 1:** Vector dynamics parameters.

Probability to move from twig to twig	*p*_*tt*_ = 0.7
Probability to move from branch to branch	*p*_*bb*_ = 0.35
Probability to move from tree to herb	*p*_*th*_ = 0.005 (tender sprouts)
	*p*_*th*_ = 1 (hard sprouts)
Probability to move from herb to tree	*p*_*ht*_ = 1 (tender sprouts)
	*p*_*ht*_ = 0 (hard sprouts)

We repeate the above steps *N*_P_ times, where *N*_P_ is the current number of prey. Step 3 is effective only if the chosen insect is a Ps and not an alternative prey. The number of infected tertiary branches is acquired every step.

## Results

### Feeding experiment

**Fig 4:**
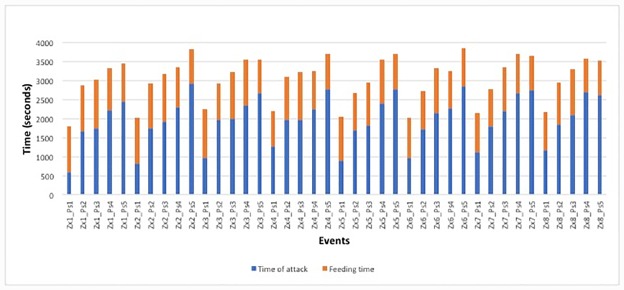
Total time per killed Ps, *τ*_tot_.

The attack and feeding time for each of the 40 observed events sustained by 8 Zr, belonging to different breeding lines and preying on five Ps simultaneously introduced into the arena, are reported in Fig 4.

The figure clearly shows the effect of the predator starving period before the experiment: for each predator, the total time for killing the first prey was significantly shorter than the following ones, and the total time per killed prey *τ*_tot_ increased during the experiment. The behavior of different predators was quite homogeneous: the relative error on the value of *τ*_tot_, per order of predation, turned out to be always ≤7.1%, as shown in Table 2.

**Table 2:** Mean values and percentage errors of the total time per killed vector from the feeding experiment.

Order of predation	*τ*_tot_ (min)	Percentage error
*Ps*_1_	34.6	7.1%
*Ps*_2_	47.8	4.7%
*Ps*_3_	53.2	4.6%
*Ps*_4_	57.4	5.0%
*Ps*_5_	61.0	4.0%

From the experimental data, the average time occurred between one feeding act and the following one turned out to be 50.8 minutes with a standard deviation of 9.6 minutes, divided in (32.9±10.4) minutes for the time of attack and (17.9±2.1) minutes for the feeding time. The incertainity on the time of attack (32%) was significantly higher than the one on the feeding time (12%). For some of the predators, the time of attack on the first prey turned out to be 2*σ* away from the mean value. This circumstance suggested to consider the hypothesis of rejecting the first data for each predator, in order to discuss the worst case. With such a choice the mean time and standard deviations for *τ*_tot_ turned out to be (54.8±5.5) minutes.

Experimental data were used to fix the value of the parameter *τ*_f_ in the numerical experiments. We considered simulations giving *τ*_tot_ spanning from 50 to 60 minutes, the corresponding best fit values for *τ*_f_ spanning from 40 to 51 minutes.

### Numerical simulations

We considered 3 different values of distance between centres of adjacent trees, *s* = 4, 5, 6, ranging from intensive to extensive olive orchard: keeping fixed the number of trees in the orchard in the three cases, the tree/ha density decreases with *s*. We also assumed the number of vectors to depend only on the extension of the orchard, and not on the spacing among trees; thus the initial number of vector/ha was assumed to be the same in the three cases. These choices correspond to have an orchard surface and a vector number increasing with *s*. For this reason, as already discussed in [1], intensive orchards, with smaller distance between neighbour trees, *s* = 4, are favoured with respect to the sparse ones, *s* = 6: higher distances among trees correspond to higher numbers of vector/tree, resulting in an increased risk of each tree to get infected. We confirm the same finding in the present work.

**Fig 5:**
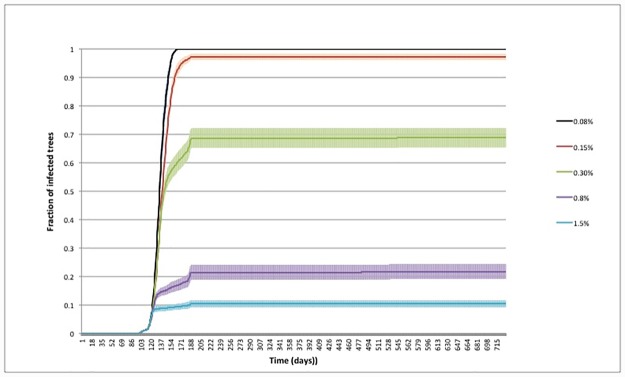
Closed orchard—Prevalence of infection as function of time and number of predators per prey. Spacing *s* = 6; total time per killed prey *τ*_tot_ = 50 min.

**Fig 6:**
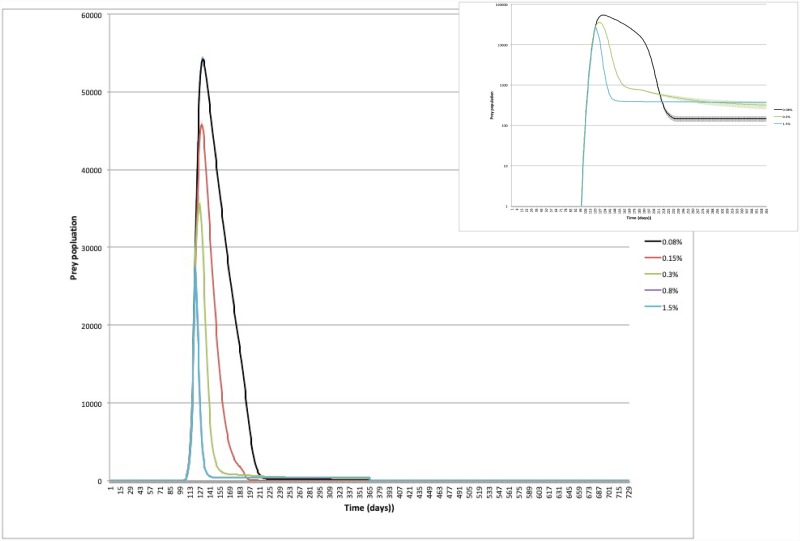
Closed orchard—Time evolution of prey for different number of predators per prey. Spacing *s* = 6; total time per killed prey *τ*_tot_ = 50 min.

In Figs 5 and 6, we plot the time evolution of the infected tree fraction and of the prey number, respectively, in the case of spacing *s* = 6, for the closed system. As we see, the infection was essentially arrested within the first year, although a finite number of survived insects was still present at the hardening of the trees (see Fig 5). However the subsequent mechanical action on the egg and juvenile stages effectively reduced the vector population to few units at the second year, so that a further inundation with Zr annihilated the vector population in a very short time window.

The time evolution of prey revealed interesting aspects. In the inset of Fig 6 the prey population is plotted in logarithmic scale. A one step decay in the number of prey was observed when the number of predators was large; a two step decay was observed when the number of predators was small. At intermediate values a logarithmic tail was observed at long time. We interpreted this different dynamics as an effect of the different relation among the number of prey per tree (*ρ*_*PT*_ = *N*_*P*_/*N*_*tree*_) and the number of prey per predator (*ρ*_*PZ*_ = *N*_*P*_/*N*_*Z*_). Assuming that predators were randomly distributed in the orchard at the inundation time, if *ρ*_*PZ*_ ≪ *ρ*_*PT*_, each predator essentially consumed its own food stock on the same tree. The prey population was annihilated without the need for predators to move from tree to tree. For *ρ*_*PZ*_ ≫ *ρ*_*PT*_, after consuming the prey on the first explored tree, each predator could easily find a nearest neighbour tree, unexplored by other predators, where searching for food. For comparable values of *ρ*_*PZ*_ and *ρ*_*PT*_, after killing the prey on the first explored tree, each predator moved to other trees, but in so doing it experienced the competition with other predators. The spatial prey distribution on trees could become fractal and thus predators need more time to find prey. This process caused the long tail observed in the data at intermediate *N*_*Z*_ and, consequentially, a counterintuitive delay in the annihilation of the prey population with respect to smaller values. Moreover, for the same reason these values of *N*_*Z*_ presented the maximum dispersion in the prevalence of infection, as shown in Fig 5.

**Fig 7:**
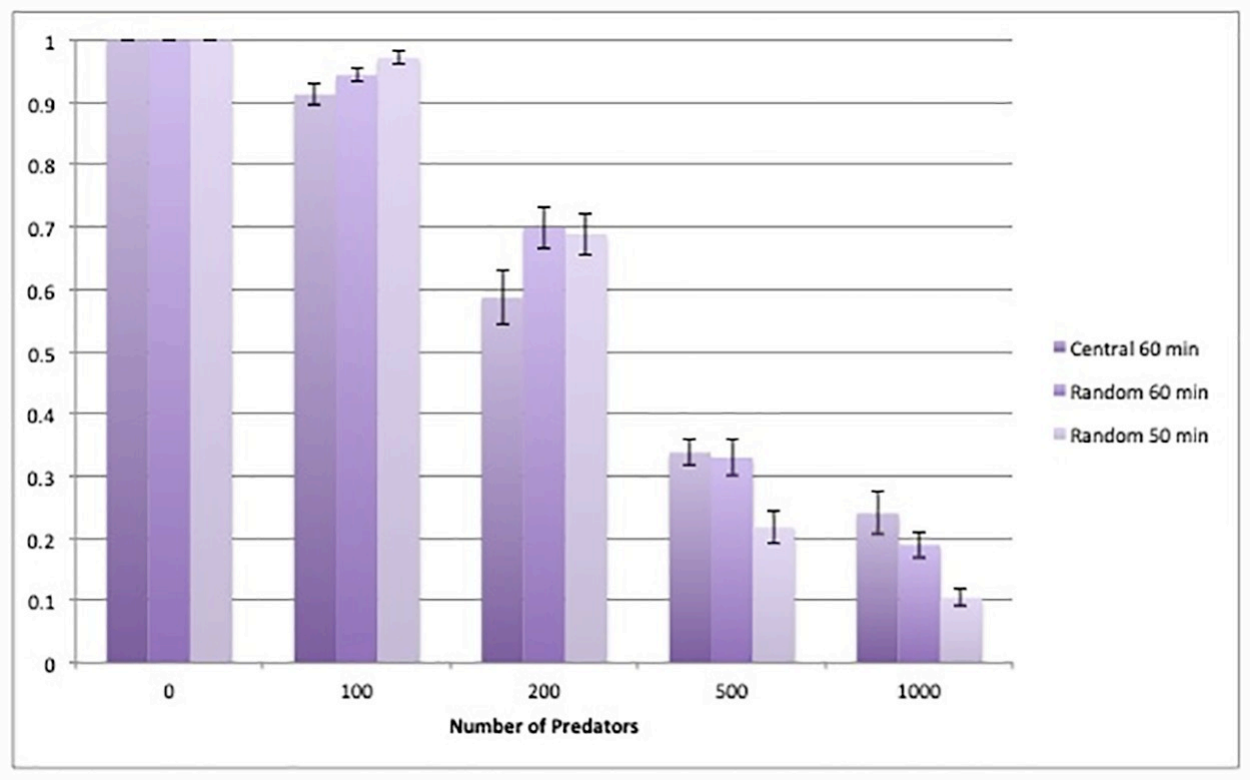
Closed orchard—Prevalence of infection for two different values of total time per killed prey (*τ*_tot_ = 50 min and 60 min) and two different release procedures.

The minimum time from the last feeding, *τ*_f_, in the range here considered, did not significantly affect the infection prevalence (as we see in Fig 7). Similarly, no significant difference within the errors, were observed when the predators were released all together at the eclosion time in the centre of the orchard, instead of being randomly distributed in the orchard (see also Fig 7).

**Fig 8:**
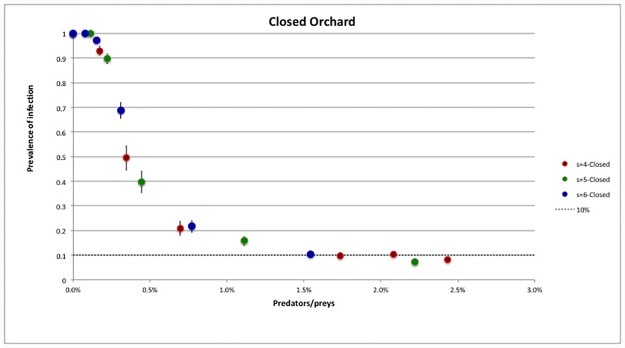
Closed orchard—Prevalence of infection after the second year as function of the number of predators per prey, *N*_z_/*N*_P_. Total time per killed prey *τ*_tot_ = 50 min.

In Fig 8 a perfect scaling was obtained by plotting the prevalence of infection as a function of the number of predators per prey, 1/*ρ*_*PZ*_, pointing out that this is the relevant parameter for the pest management. We have also seen that a proportion of roughly 2.25% predators per prey was always enough to decrease the prevalence of infection, after the second year, below 10%.

**Fig 9:**
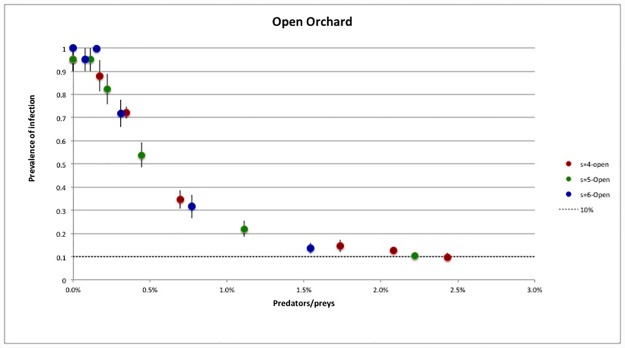
Open orchard—Prevalence of infection after the second year as function of the number of predators per prey, *N*_z_/*N*_v_. Total time per killed prey *τ*_tot_ = 50 min.

A similar scaling was obtained in the open system (see Fig 9). Comparing the two cases, we have seen that the decreasing of infection prevalence was slower than in the closed case: as expected, arresting the infection in the open orchard was more complex than in the isolated system. Interestingly, also in this case, the relevant parameter governing the spreading of the disease is the number of predators per prey present in the orchard.

The scaling behaviour turned out to be stable with respect to variation of the prey population. Numerical experiments with a prey population doubled with respect to the one considered here (as for instance in case of partial failure of treatments on egg and juvenile stages), showed that the proportion of predators per prey necessary to reduce the prevalence of infection to the 10% remained unchanged (i.e. roughly 2.25%).

**Fig 10:**
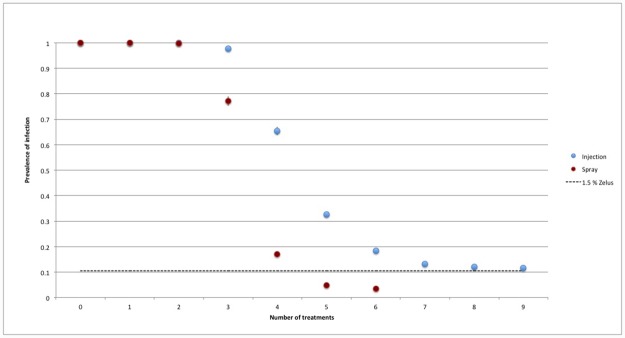
Closed orchard—Comparison among biological and chemical treatments. Spacing *s* = 6. Total time per killed prey *τ*_tot_ = 50 min.

Comparing the efficacy of the biocontrol action proposed in the present paper with the one obtained with the chemical treatments discussed in [1] (see Fig 10), it clearly appeared that the biocontrol allowed to reach the same threshold efficacy (10%) of repeated injection treatments, with the use of a very low percentage of predators per prey. But the use of Zr turned out to be less effective then spray treatments, which exhibited a threshold efficacy of 3.3%. The same results was obtained for every spacing and for the open system as well.

**Fig 11:**
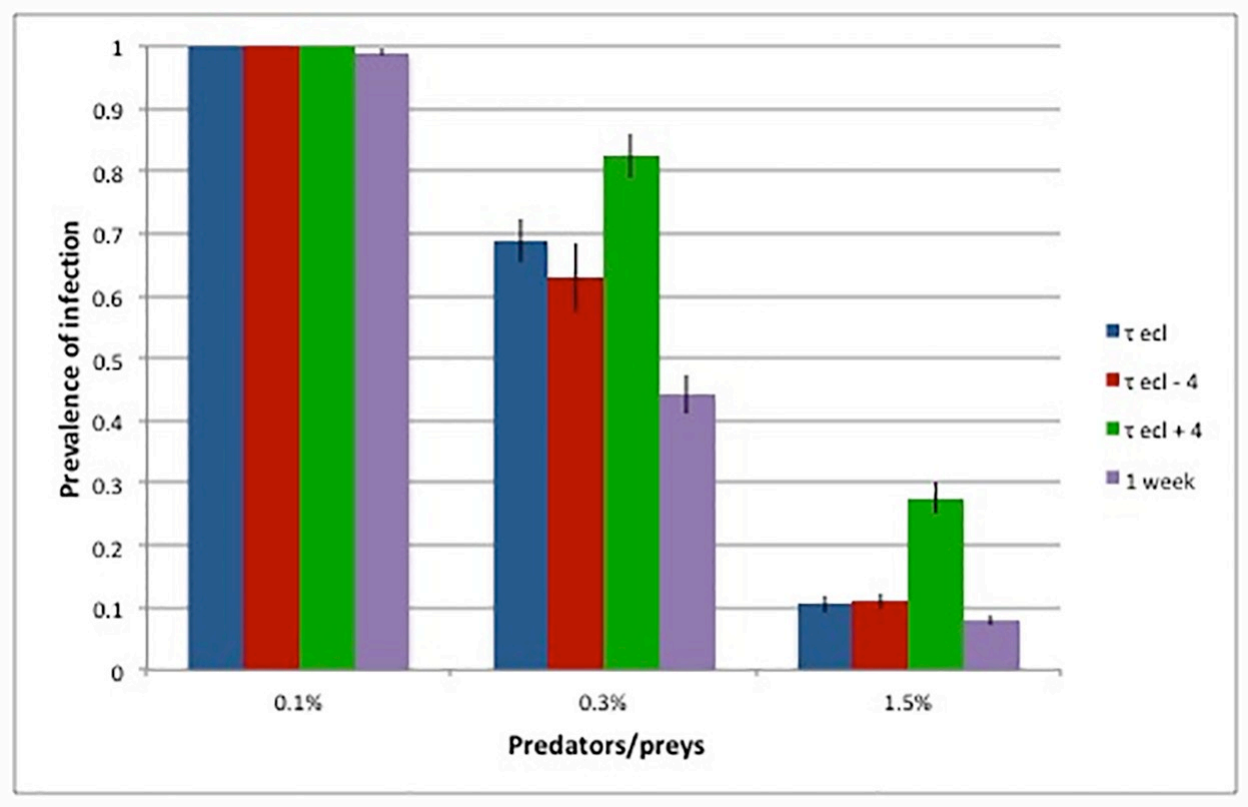
Closed orchard—Effect of anticipating or postponing the Zr introduction of 4 days with respect to the mean eclosion time. Spacing *s* = 6. Total time per killed prey *τ*_tot_ = 50 min.

Finally, notice that, as for the chemical treatments, the timing of the inundations was fundamental: if the first treatments against adults were delayed with respect to the eclosion time, they were completely ineffective for the transmission control, although they could lead to the annihilation of the adult population. Fig 11 shows that even a very short delay (4 days in the example), significantly reduced the efficacy of the inundation measure: the prevalence of infected trees increased from 10% to 28% in the case with 1/*ρ*_*PZ*_ = 1.5%. On the contrary, anticipating the inundation of 4 days did not improve the efficacy of the treatment. An interesting and efficient alternative consisted in introducing the predators not all at the same time (i.e. at the expected mean eclosion day), but distributed during a one-week period centred on the eclosion mean time. As shown in Fig 11, this solution allowed to achieve even better results, in that predators could intercept prey eventually appearing few days before the mean eclosion time. The efficacy of this distributed release procedure allowed to reduce the impact of eventual uncertainties in the forecasted eclosion time.

## Discussion

In the present paper, we have shown that, with a modest number of Zr, it is possible to contain the *Xylella* invasion below a tolerable threshold. For instance, within our schematization and choice of relevant parameters, assuming as in [1], a stable population of Ps equal to 1.000.000/ha, that reduces to 90.000/ha adult individuals after egg and juvenile treatments, an inundation with roughly 4.000 predators/ha is sufficient to reduce the overall prevalence of infection to the 10% in two years.

We remark that the role of Zr in controlling the spreading of the bacterium is double-fold: on one side it targets the adult before or at their first transmission (plant infection), on the other side it reduces the actual vector population size, firstly, impeding the female killed during the first inundation to lay eggs, and secondly minimizing the generation of vectors born by the survived females during the second inundation.

There are moreover many other arguments in favour of Zr. Adults of Zr attack actively moving prey [35], [59], [60], (see S5 and S6 Figs) showing a functional response to prey abundance [36], [61], prey size [62], [63] and positive chemotaxis to honeydew of Hemiptera (see S7 Fig). We suggest to inundate the orchard by adult Zr, and not pre-adult, because adults only are able to handle adult vectors attacking them successfully. Cannibalism among Zr juveniles or adults is frequent if starvation occurs [59], eventually leading to self-control population. Occasionally *Zelus* may bite humans for self-defence, if roughly managed and accidentally or intentionally squeezed. Finally, Zr does not grow feeding on plant or plant parts [64], being able, in its juvenile instar, to survive for a while only, feeding on pollen or on sweet substances from plant nectaries [65].

Recent evidences suggest that cicadas are not implicated in Xf infection or transmission [66]. This study denies the existence of such big *Xylella* vectors, so big to be out of the Zr prey range and not manageable by the predator. On the same token, it suggests that the rest of the actual vectors, or candidates, fall into the available prey size for Zr.

A further argument in favor of the use of Zr consists in the evidence that it also preys on several relevant olive-frequenting insects and that many of them are olive pests. Here we just note that many antagonists of olive pests are below (or at the limit of) the adult Reduviidae predation range and considerably more motile. Further the probability that the Zr will encounter olive fly or moth caterpillar adults is larger than the probability to encounter small or very small *Metaphycus* (Hymenoptera: Encyrtidae), *Pnigalio* (Hymenoptera: Eulophidae) or *Lasioptera berlesiana* (Paoli, 1907).

In [67] authors describe an experiment in which Zr and other predators, crysopid and presumed-to-be-shared-prey (the aphids) were put together within closed cages. They showed that Zr and other predators [68] have a strong positive prey-size functional response versus the Crysopid and a strong negative prey-size functional response to the aphids. Zr action, thus, resulted in intra-guild predation and consequent aphid outbreak. Further Rosenheim papers [69] and [70] recognize the Zr preferences in caging experiments and demonstrates that Zr is not a generalist predator, but it is constrained by ontogenetic and taxonomic preferences.

One might wonder if the evaluation of predation parameters, as well as the efficacy test of the inundation procedure through numerical simulation, could have been better achieved through an in-field experiment. Quantitative population dynamic during transitory events is among the most complicated phenomena to describe in insect control. The evaluation of Xf vectors and transmission to olive trees are not an exception. This is because the minimum and maximum accuracy of the sampling method and the sampling error may change from sample to sample during the observation due to changes in the observed insect behavior. This makes quantitative sampling techniques [30], [31] unavailable in the case of Italian Xf adult vectors.

Moreover in [71], authors suggest the use of simulations by a cohort life table, HTL, or MDLT, to quantify the biocontrol effect before going in the field, because of the inadequacy of monitoring procedures and the consequent unpredictability of indirect effects [32]. In particular the otherwise available techniques [33] are not appropriate for this kind of study, as shown in [67], [68], [69] where different results are obtained by comparing the effect of Zr action on in-cage plants and surrounding plants. In [70], authors admit that *Zelus* is not a generalist predator and the cage influences the experimental results. Moreover, even wishing to try a semi-field experiment, we encounter some inextricable difficulties in experiment setup that we can resume in a single question: how to imitate a time and space open inundation phenomenon within a time and space limited artificial context? Actually the predator/prey ratio in a limited cage or mesocosm environment will trigger the result in a predictable way, unacceptably biasing the experiment. Being impossible to evaluate the *Zelus* efficacy in a semi-field experiment for the infection control by measuring killed vector, the only way to evaluate the predator efficacy would be to search Xf and test the experimented olive plants, even if in a space limited condition. The latter means to isolate and check, by plate culture or molecular methods, all the caged and non-caged olive branches or all the olive plants into the mesocosm. The tests, destructive, will last for many month or years after the experiment. Alternatively we may wait for symptoms that will eventually appear years later [72]. In synthesis, we abandoned the idea of the semi-field test because multiple factors would act at the same time and in the same place on the experiment, biasing the collections of the results in a way impossible to clarify. The clean predation experiment joined with the numerical simulated experiment that we proposed is the most similar and repeatable way to simulate the field condition, in our opinion, and by the time.

Our model presents some limits. First of all, it is not realistic when twigs belonging to a main olive branch are considered situated in a single point of the lattice, neglecting their linear dimension. In principle, this approximation reduces the prey-predator encounter time. However, the possible decrease of the prey-predator encounter time due to our schematization is counterbalanced by having put a minimum time from the last feeding that allows the attack and feeding time observed in the experiments to be reproduced in the simulations. We propose to better quantify the effect of these approximations in future.

In our numerical experiments, we put the ratio between the number of other prey and the total number of prey, R, equal to 0.5. We verified that the infection prevalence does not change within the errors by keeping fixed the number of Ps and by varying the number of other prey with *R* spanning from zero to 0.65 (the number of predators is kept fixed too). Our predictions are thereby very solid in a wide range of R values. On the other hand, a modest increase of the infection prevalence is observed by spanning R from 0.65 to 0.8. Moreover, in the limit R → 1, in which Ps only becomes a negligible fraction of the total prey, predators are expected to mostly feed on other prey, while Ps, unhindered, continues to spread the disease, making the proposed strategy, to inundate the olive orchard by means of Zr against Xf spreading, totally ineffective. For an improved modelization and an increased capacity of most realistic forecast, it is then crucial to quantify the real value of R in olive orchard, and to evaluate, in simulations, the effect of its variation in the whole realistic range of variability.

As already mentioned, in literature there are few scattered information about possible natural enemies of Ps for application in biological control. We discuss some proposals and explain why we do not trust them as possible alternatives to Zr.

- Di Serio et. al. [22] reports an entomopathogenic epizootic over Ps juveniles bred in the mesocosm set in Turin. The described fungal intensive natural biocontrol event in mesocosm was sustained by *Beauveria bassiana* (Balsamo) Vuillemin, 1912) and *Fusarium oxysporum* (von Schlechtendal, 1824). Both fungi were naturally present in the environment. Similar events are never or quite scarcely observed in nature and we guess that the case in Turin mesocosm was facilitated by high relative humidity reaching the dew point with consequent water condensation over the juveniles-hosting herbs. The former circumstance was facilitated by low air-flow exchange with outer environment, also due to the limited volume of the mesocosm. Both conditions are unrealistic in nature.
- Data about *Verrallia* spp. (Mik, 1899) reveal a parasitoidism on Ps in the range 18–46%, allowing [73] to suggests that *Verrallia* parasitizes the vector, with a positive functional response to available host individuals and species relative abundance. However the same authors admit that this result may be biased by the collection technique of the parasitized individuals. We add that parasitized individuals shall be more readily caught because the are slowed and overweighted by the presence of parasitoid in their distended abdomen. Moreover, the studies do not use any proven quantitative adult sampling method, and data of parasitism do not refer to the host population, but to the number of collected adults only. Wishing to manage the Xf invasion by the infection control, one should consider that the parasitized individuals act as healthy individuals on the side of plant pathogen transmission, i.e., they are still able to acquire Xf and to transmit the pathogen to hitherto uninfected plants, until they die. Furthermore, even if the adult vector population is *Verrallia*-zombified because the parasitic castration leads reproductive organs to atrophy [73], *Verrallia* is not so effective to reduce the overall vector population of the following year. Furthermore, we miss any mass-breeding option for *Verrallia* to inundate olive orchards, as we miss for *Ooctonus vulgatus* (Haliday, 1833) [74]. Finally, olive management in conventional or organic IPM still needs occasional chemical action that will disrupt the *Verrallia* action, admitting it exists, in the environments of the Oleo-Ceratonion siliquae Mediterranean vegetation zone.

A final comment on the usage of satellite imagery to contain the pest: in our opinion OQDS symptoms gathered by satellite will never serve to prevent already occurred Xf infection, differently from the claim of [2]. By the time it is impossible to detect a new infection promptly (we mean in 15-20 days from the first transmission, an interval useful to impose a control action) and it will rest impossible to detect it for a long time in future, eventually. The available studies by remote sensing all detect the symptoms that occur years after the infection and are unavailable to prevent the already occurred infection, obliviously. The only tool to avoid or prevent the infection is the control of the vectors, timely applied before the first transmission.

## Conclusion

To conclude, in this study we presented the encouraging results of a work based on the integration of a laboratory experiment with a numerically simulated experiment, demonstrating the potential efficacy of an inundation strategy with a natural enemy of the Xf main vector, Zr, for arresting the OQDS syndrome that is devastating the fruit orchard production in Italy. The laboratory experiment showed that Zr presents itself as a good candidate to conceive a biocontrol strategy against the Xf vector and infection. The experiment was used to understand the feeding dynamics and to measure the killing time per prey, in order to parameterize a numerical experiment for simulating the action of *Zelus renardii* predator in an olive orchard. We showed, through numerically simulated experiments, that an inundative biological control action carried out with a very small number of Zr can arrest new infection and reduce the transmission of the Xf below an acceptable threshold in two years of control. In particular the pest containment, achieved with the biological strategy proposed, showed considerable positive points in comparison with the one obtained through any sequence of repeated injections of insecticides. Its efficiency is comparable with the one obtained in one year with 4 or 5 spray treatments, but with a clear reduction of the environmental impact. The possibility to avoid massive use of synthetic chemical insecticides, that cause concern among producers and consumers, adopting an efficient and “green” solution to the Xf invasion, should convince the policy makers to allow an in-field validation of this procedure.

## Supporting information

S1 Fig *Zelus renardii* empty choria on Citrus infested by *Aleurocanthus spiniferus* & *Aleurothrixus floccosus*. (PDF)

S2 Fig A *Zelus renardii* laying eggs. (PDF)

S3 Fig Mass collection and storage of adult *Philaenus spumarius* by proper low-stress devices. (PDF)

S4 Fig A shot from one of the very early observations on the preferred *Zelus renardii* prey. (PDF)

S5 Fig *Zelus renardii* preying a fly on Citrus. (PDF)

S6 Fig a. A *Zelus renardii* najad preying Drosophila larva. b. A *Zelus renardii* najad preying Drosophila adult.
(PDF)

S7 Fig *Zelus renardii* preying *Harmonia axyridis* (Coccinellidae) adult. (PDF)

S1 File (PDF)

S2 File (PDF)

S3 File (PDF)
