# Supplementary figures and images for: A biological control model to manage the vector and the infection of Xylella fastidiosa on olive trees
Source: PLoS One. 2020 Apr 30;15(4):e0232363. doi: 10.1371/journal.pone.0232363 (PMC7192417; doi:10.1371/journal.pone.0232363)

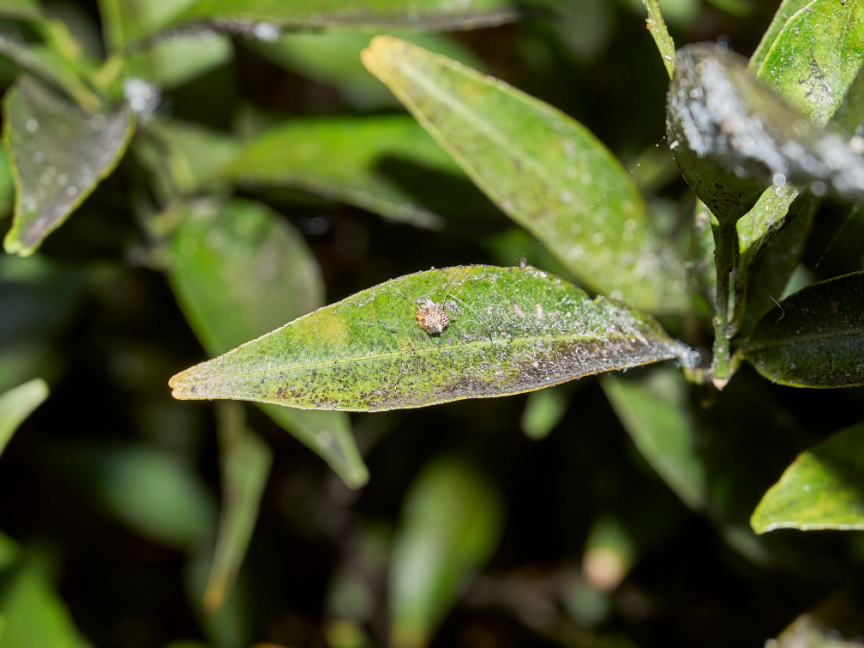

Supplement: S1 Fig — (PDF) [file pone.0232363.s001.pdf]

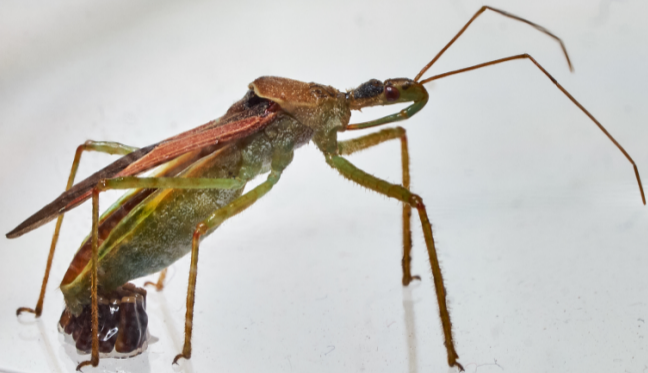

Supplement: S2 Fig — (PDF) [file pone.0232363.s002.pdf]

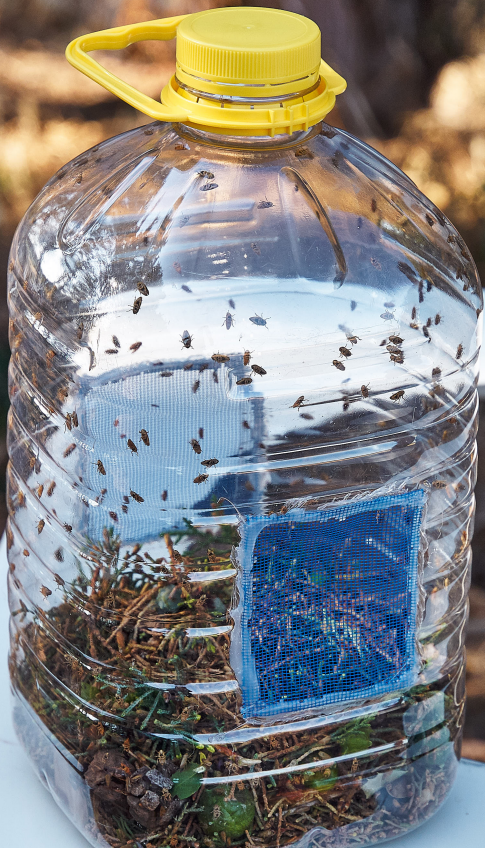

Supplement: S3 Fig — (PDF) [file pone.0232363.s003.pdf]

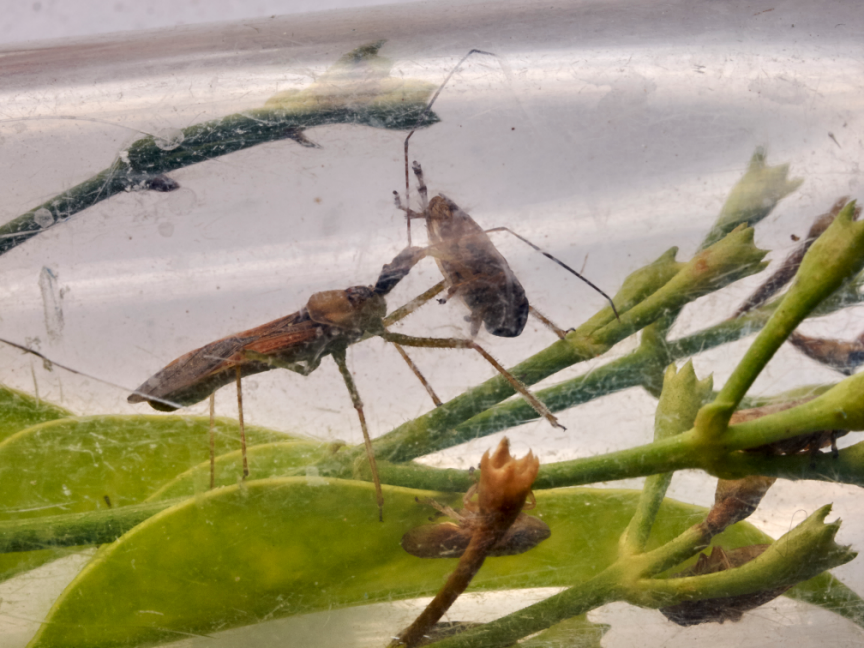

Supplement: S4 Fig — (PDF) [file pone.0232363.s004.pdf]

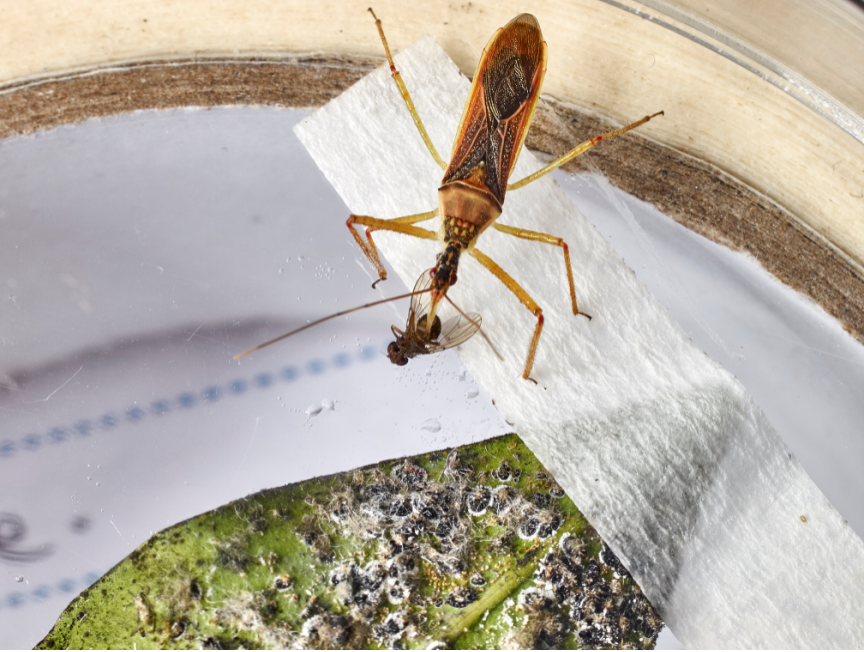

Supplement: S5 Fig — (PDF) [file pone.0232363.s005.pdf]

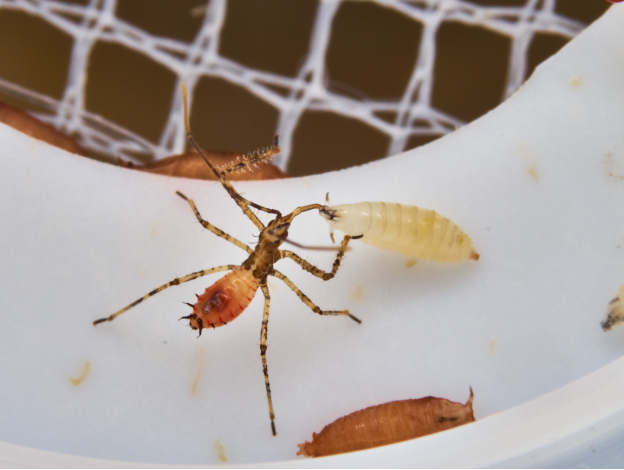

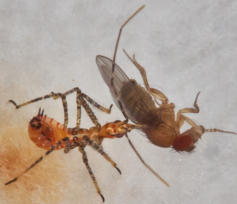

Supplement: S6 Fig — a. A Zelus renardii najad preying Drosophila larva. b. A Zelus renardii najad preying Drosophila adult. (PDF) [file pone.0232363.s006.pdf]

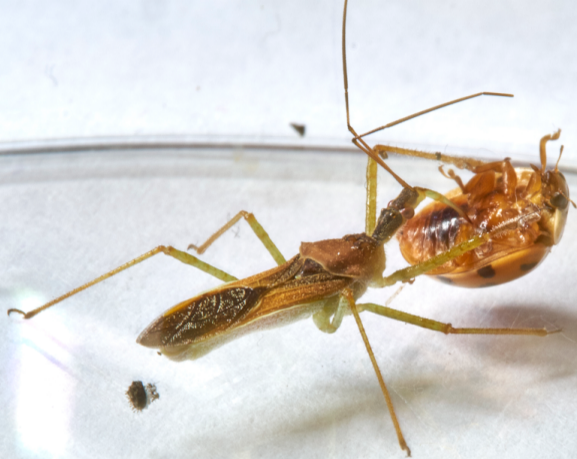

Supplement: S7 Fig — (PDF) [file pone.0232363.s007.pdf]
