# Supplementary material for: A biological control model to manage the vector and the infection of Xylella fastidiosa on olive trees
Source: PLoS One. 2020 Apr 30;15(4):e0232363. doi: 10.1371/journal.pone.0232363 (PMC7192417; doi:10.1371/journal.pone.0232363)
Supplement: S1 File — (PDF) [file pone.0232363.s008.pdf]

Order	Family	Species	Reference	Approach	Experiments
Blattodea	Blattidae	*Periplaneta americana* (Linnaeus, 1758)	Davranoglou, 2011	Qualitative	Lab
Orthoptera	Gryllidae	*Acheta domesticus* (Linnaeus, 1758)	Davranoglou, 2011	Qualitative	Lab
Thysanoptera	Thripidae	*Scirtothrips citri* (Moulton, 1909)	Horton, 1918a	Qualitative	Lab
			Horton, 1918b	Qualitative	Lab
Hemiptera	Pseudococcidae	*Pseudococcus citriculus* Green, 1922	Ambrose, 2003	Qualitative	Lab
	Psyllidae	*Glycaspis brimblecombei* Moore, 1964	Garrison, 2001	Qualitative	Field
		Macrohomotoma gladiata Kuwayama, 1908	Cornara *et al*., 2016	Quantitative	Field
		Heteropsylla cubana Crawford, 1914	D'Hervé *et al*., 2018	Qualitative	Field
	Liviidae	*Diaphorina citri* Kuwayama, 1908	Barrera *et al*., 2010	Qualitative	Field
			Pardo-Melgarejo and Miranda-Salcedo, 2016	Qualitative	Field
	Aphididae	*Therioaphis maculata* (Buckton, 1899)	Nielson and Henderson, 1959	Qualitative	Field
		Aphis gossypii Glover, 1877	Kessing and Mau, 1991	Qualitative	Field
			Rosenheim *et al*., 1993	Quantitative	Field
			Rosenheim *et al*., 1995	Quantitative	Field
			Cisneros and Rosenheim, 1997	Quantitative	Field
		Brachycaudus tragopogonis (Kaltenbach, 1843)	Barrera *et al*., 2010	Qualitative	Field
		Brachycaudus persicae (Passerini, 1860)	Potin Téllez, 2008	Quantitative	Lab
		Macrosiphum pisi (Harris, 1776)	El-Tom, 1965	Qualitative	Lab
		Macrosiphum (Macrosiphum) rosae (Linnaeus, 1758)	Barrera *et al*., 2010	Qualitative	Field
		Macrosiphum euphorbiae Linnaeus, 1758	Potin Téllez, 2008	Quantitative	Lab
		Myzus persicae (Sulzer, 1776)	Potin Téllez, 2008	Quantitative	Lab
			Barrera *et al*., 2010	Qualitative	Field
		Therioaphis maculata (Buckton, 1899)	Nielson and Henderson, 1959	Qualitative	Field
	Cicadellidae	*Carneocephala* spp.	El-Tom, 1965	Quantitative	Lab
		Draeculacephala spp.	El-Tom, 1965	Quantitative	Lab
		Acinopterus spp.	El-Tom, 1965	Quantitative	Lab
		Aceratagalia spp.	El-Tom, 1965	Quantitative	Lab
	Dictyopharidae	*Dictyophara europaea* (Linnaeus, 1767)	Davranoglou, 2011	Qualitative	Lab
	Delphacidae	*Perkinsiella saccharicida* Kirkaldy, 1903	Muir, 1921	Qualitative	Field
			Nishida, 1955	Qualitative	Field
			Ali, 1978	Qualitative	Field
			Weirauch *et al*., 2006	Qualitative	Field
	Miridae	*Pycnoderes quadrimaculatus* Guérin-Méneville, 1857	Zimmerman, 1948	Qualitative	Field
		Lygus spp.	El-Tom, 1965	Qualitative	Field
		Lygus hesperus (Knight, 1917)	Zink and Rosenheim, 2008	Qualitative	Field
	Rhopalidae	*Liorhyssus hyalinus* (Fabricius, 1794)	Zimmerman, 1948	Qualitative	Field
	Pentatomidae	*Chlorochroa ligata* (Say, 1832)	Morril, 1910	Qualitative	Lab
		Euschislus impictiventris Stål, 1872	Clancy, 1946	Qualitative	Lab
		Nezara viridula Linnaeus, 1758	Davranoglou, 2011	Qualitative	Lab
		Eurydema spp.	Davranoglou, 2011	Qualitative	Lab
Neuroptera	Chrysopidae	*Chrysoperla carnea* Stephens, 1836	Knowlton, 1944	Qualitative	Field
			Rosenheim *et al*., 1993	Quantitative	Field
			Cisneros and Rosenheim, 1997	Quantitative	Field
			Ambrose, 2000	Quantitative	Field
			Ambrose, 2003	Qualitative	Field
Lepidoptera	Bucculatricidae	*Bucculatrix thurberiella* Busck, 1914	Tuttle *et al*., 1961	Qualitative	Lab
			Ali, 1978	Quantitative	Lab
	Gelechiidae	*Pectinophora gossypiella* (Saunders, 1844)	Orphanides *et al.*, 1971	Qualitative	Lab
	Pieridae	*Colias eurytheme* Boisduval, 1852	El-Tom, 1965	Qualitative	Lab
	Noctuidae	*Helicoverpa armigera* (Hübner, 1808)	Ewing and Ivy, 1943	Qualitative	Field
		Helicoverpa zea (Boddie, 1850)	Ali and Watson, 1978	Quantitative	Lab
		Heliothis virescens (Fabricius, 1777)	Wille, 1951	Qualitative	Field
			Ali and Watson, 1978	Quantitative	Lab
			Ables,1978	Quantitative	Lab
			Cohen and Tang, 1997	Quantitative	Lab
		Spodoptera exigua (Hübner, 1808)	Cohen and Tang, 1997	Quantitative	Lab
			Ali and Watson, 1978	Quantitative	Lab
Diptera	Drosophilidae	*Drosophila melanogaster* Meigen,1830	El-Tom, 1965	Qualitative	Lab
			Mbata *et al*., 1987	Qualitative	Lab
			Barrera *et al*., 2010	Qualitative	Lab
	Tephritidae	*Bactrocera cucurbitae* Coquillett, 1849	Severin *et al*., 1914	Qualitative	Field
			Nishida, 1955	Qualitative	Field
		Anastrepha ludens Loew, 1873	Barrera *et al*., 2010	Qualitative	Lab
		Ceratitis capitata Wiedemann, 1824	Barrera *et al*., 2010	Qualitative	Lab
Coleoptera	Coccinellidae	*Adalia bipunctata* (Linnaeus, 1758)	Barrera *et al*., 2010	Qualitative	Field
		Cryptolaemus montrouzieri Mulsant, 1850	Barrera *et al*., 2010	Qualitative	Field
		Eriopis connexa (Germar,1824)	Barrera *et al*., 2010	Qualitative	Field
		Hippodamia variegata (Goeze, 1777)	Barrera *et al*., 2010	Qualitative	Field
		Harmonia axyridis (Pallas, 1773)	Faúndez, 2015	Qualitative	Field
	Curculionidae	*Anthonomus grandis grandis* Boheman, 1843	Drees and Jackman, 1999	Qualitative	Field
Hymenoptera	Scoliidae	*Campsomeris marginella modesta* Smith, 1855	Muir, 1931	Qualitative	Field
		Scolia manilae (Ashmead, 1885)	Muir, 1931	Qualitative	Field
	Apidae	*Apis mellifera* Linnaeus, 1758	Muir, 1931	Qualitative	Field
			Nishida, 1955	Qualitative	Field
			Vivas, 2012	Qualitative	Field
	Aphelinidae	*Aphytis* spp.	Heimpel *et al*., 1997	Qualitative	Field
			Curkovic *et al*., 2004	Qualitative	Field

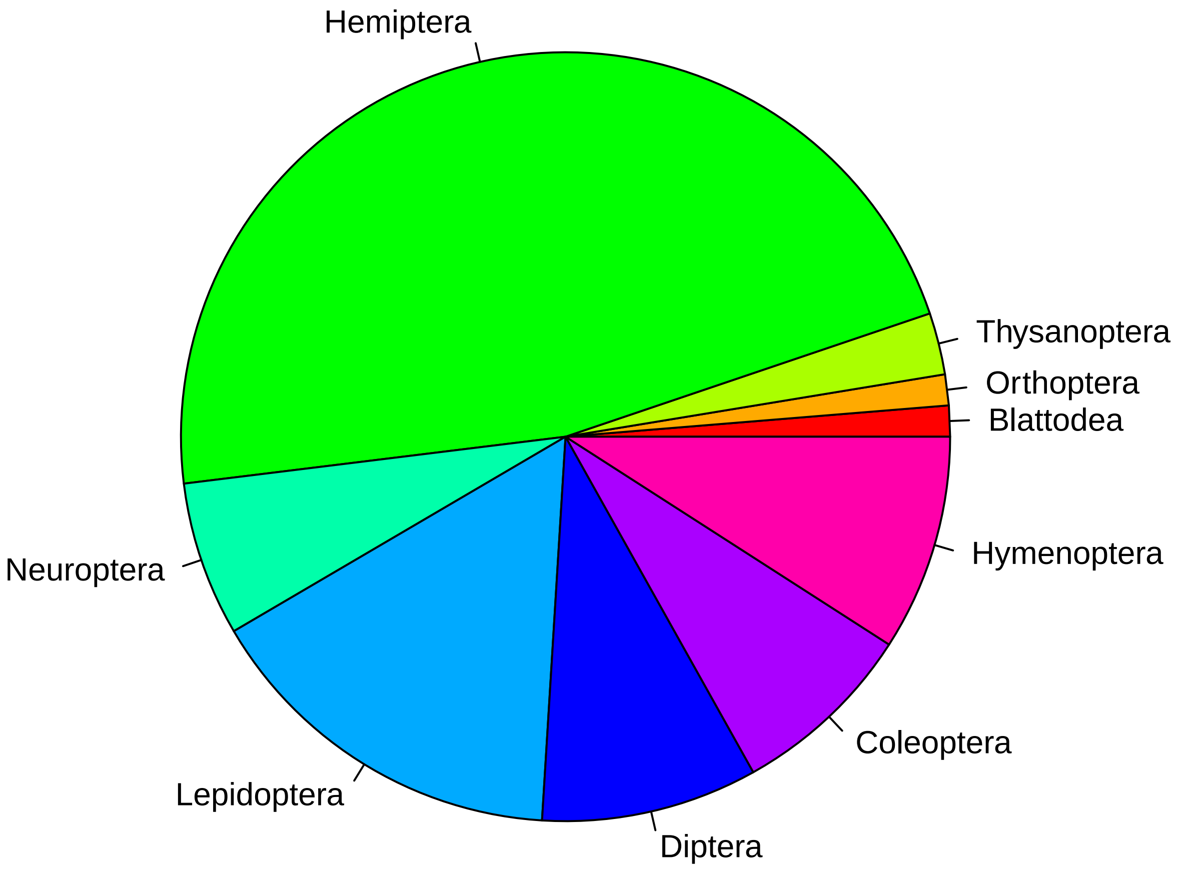

Hemiptera
Thysanoptera
Orthoptera
Blattodea
Hymenoptera
Coleoptera
Diptera
Lepidoptera
Neuroptera
