# Supplementary material for: A biological control model to manage the vector and the infection of Xylella fastidiosa on olive trees
Source: PLoS One. 2020 Apr 30;15(4):e0232363. doi: 10.1371/journal.pone.0232363 (PMC7192417; doi:10.1371/journal.pone.0232363)
Supplement: S2 File — (PDF) [file pone.0232363.s009.pdf]

Zelus renardii and Z. tetracanthus (Hemiptera: Reduviidae): biological attributes and the potential for dispersal in two assassin bug species

C Weirauch, C Alvarez, G Zhang - Florida entomologist, 2012 - BioOne

… ABSTRACT The **leafhopper assassin bug**, Zelus renardii Kolenati, is a natural enemy and stands out among species in the large New World genus Zelus Fabricius (~60 spp.) by its introduction to and establishment in 3 biogeographic regions …

Citato da 17 Articoli correlati Tutte e 10 le versioni Web of Science: 11

Three new assassin bug records (Hemiptera: Heteroptera: Reduviidae) for the Balkan Peninsula

N Simov, D Gradinarov, LR Davranoglou - Ecologica Montenegrina, 2017 - biotaxa.org

… & det. LR Davranoglou (DPC). Distribution. The native range of the **leafhopper assassin bug** Zelus renardii includes North and Central America. In the 20th century Z. renardii spread to Hawaii and other tropical areas in the Pacific Region (Weirauch et al. 2012) …

Citato da 5 Articoli correlati Tutte e 2 le versioni

New records of the Nearctic **leafhopper assassin bug**, Zelus renardii Kolenati, 1857 in the Iberian Peninsula (Hemiptera: Heteroptera: Reduviidae)

M Goula, F Lizana, A Miralles-Núñez - researchgate.net

After the first record of Zelus renardii Kolenati, 1857 in Spain in 2012, the species has been recorded in other provinces in the south of this country, and part of the information is only in the internet. In this paper, the species is reported for the first time from several spanish Pro …

First record of the nearctic Zelus renardii (Heteroptera, Reduviidae, Harpactocorinae) in Europe

PV Petrakis, P Moulet - Entomologia Hellenica, 2011 - ejournals.epublishing.ekt.gr

… KEYWORDS: Zelus renardii, distribution, invasive species. Introduction The **leafhopper assassin bug** Zelus renardii Kolenati, 1857 (Hemiptera, Reduviidae, Harpactocorinae) was found in three localities in Attica, Greece for the first time in Europe …

Citato da 20 Articoli correlati Tutte e 5 le versioni

Supplementary Material for" Zelus Renardii and Z. Tetracanthus (Hemiptera: Reduviidae): Biological Attributes and the Potential for Dispersal in two Assassin Bug …

C Weirauch, C Alvarez, G Zhang - 2012 - journals.flvc.org

Zelus renardii (Kolenati, 1856), a new world reduviid discovered in Europe (Hemiptera: Reduviidae: Harpactorinae)

LR Davranoglou - Entomologists Monthly Magazine, 2011 - researchgate.net

… Zelus renardii (Kolenati, 1856) commonly called the '**Leafhopper Assassin Bug**' is widely distributed in the New World, being recorded from the continental USA (California and adjacent states and New Mexico), Hawaii, western and southeastern Mexico, Guatemala, Jamaica …

Citato da 19 Articoli correlati Tutte e 3 le versioni

REPRODUCTIVE BEHAVIOR IN ZELUS RENARD/I KOLENATI 1857 (HEMIPTERA: REDUVIIDAE)

KJ Mbata, ER Hart, RE Lewis - MAR I 10A4, 1987 - lib.dr.iastate.edu

… INTRODUCTION The geographic distribution of the **leafhopper assassin bug**, Zelus renardii Kolenati, ranges from the tropical regions of Central America, where it is sympatric with a close relative Z. cervicalis Stal, northward to the warm temperate regions of Texas and …

Citato da 3 Articoli correlati Tutte e 2 le versioni

Field guide to predators, parasites and pathogens attacking insect and mite pests of cotton: recognizing the good bugs in cotton

AE Knutson, J Ruberson - Texas FARMER Collection, 2005 - oaktrust.library.tamu.edu
… C-7" 40 Harmonia or Asian Lady Beetle 42 Convergent Lady Beetle 44 Pink Spotted Lady Beetle 46 Collops Beetles 48 Hooded Beetle 50 Striped Earwig 52 Rove Beetles 54 Damsel Bugs 56 Spined Soldier Bug 58 Spined Assassin Bugs 62 **Leafhopper Assassin Bug** 64 Big …
Citato da 4 Articoli correlati Tutte e 9 le versioni

First record of the alien exotic sap beetle Phenolia (Lasiodites) picta (Coleoptera: Nitidulidae) in Greece

K Kalaentzis, A Mpamnaras… - ENTOMOLOGIA …, 2019 - ejournals.epublishing.ekt.gr
… 2009). Several non-native insects have also been reported in Greece over the last years, as a result of climatic change and/or intensified international movement of horticultural plants and products, eg **leafhopper assassin bug**, Zelus renardii Kolenati, 1856 or armadillo weevil …
Citato da 1 Articoli correlati Tutte e 2 le versioni

First record of Zelus renardii Kolenati (Heteroptera: Reduviidae: Harpactorinae) in Israel

T van der Heyden - Revista Chilena de Entomología, 2018 - biotaxa.org
… Zelus renardii Kolenati, 1856, commonly known as the **Leafhopper Assassin Bug**, is the only species within the New World genus Zelus Fabricius, 1803 that has been introduced to Europe … Netanel, O. (2018) **Leafhopper Assassin Bug** (Zelus renardii) …
Articoli correlati Tutte e 6 le versioni

ZELUS RENARDII (KOLENATI, 1856)

LR DAVRANOGLOU - The …, 2011 - Entomologist's Monthly Magazine …
Articoli correlati

Insect fauna of cultivated guayule, Parthenium argentatum Gray (Campanulatae: Compositae)

JD Stone, JN Fries - Journal of the Kansas Entomological Society, 1986 - JSTOR
… seriatus (Reuter) Rhinacloa forticornis Reuter Spanagonicus albofasciatus (Reuter)?whitemarked fleahopper Sixeonotus areolatus Knight Nabidae Nabis alternatus Parshley?western damsel bug Reduviidae Zelus renardii Kolenati?**leafhopper assassin bug** Z. socius (Uhler …
Citato da 11 Articoli correlati Tutte e 3 le versioni Web of Science: 7

Biología y método de cría de Zelus renardii (Hemiptera: Reduviidae), enemigo natural de Diaphorina citri (Hemiptera: Psyllidae)

JF Barrera, J Gómez-Ruiz, J Herrera-Muñoz - … sobre investigación para el manejo del …, 2010
Citato da 3 Articoli correlati

Obliquebanded leafroller.

WJ Bentley, KR Day - Obliquebanded leafroller., 2010 - cabdirect.org
Cookies on CAB Direct. Like most websites we use cookies. This is to ensure that we give you the best experience possible. Continuing to use www.cabdirect.org means you agree to our use of cookies. If you would like to, you can learn more about the cookies we use …
Citato da 1 Articoli correlati Tutte e 2 le versioni

Spider mites.

ET Natwick - Spider mites., 2010 - cabdirect.org
Cookies on CAB Direct. Like most websites we use cookies. This is to ensure that we give you the best experience possible. Continuing to use www.cabdirect.org means you agree to our use of cookies. If you would like to, you can learn more about the cookies we use …
Tutte e 2 le versioni

Omnivorous leafroller.

WJ Bentley, KR Day - Omnivorous leafroller., 2009 - cabdirect.org
Cookies on CAB Direct. Like most websites we use cookies. This is to ensure that we give you the best experience possible. Continuing to use www.cabdirect.org means you agree to our use of cookies. If you would like to, you can learn more about the cookies we use …
Tutte e 2 le versioni

Influence of non-crop plants on stink bug (Hemiptera: Pentatomidae) and natural enemy abundance in tomatoes

CG Pease, FG Zalom - Journal of Applied Entomology, 2010 - Wiley Online Library

… The following generalist predators were monitored: minute pirate bug (Orius tristicolor [White]), Nabis spp., Geocoris spp., **leafhopper assassin bug** (Zelus renardii Kolenati), spined assassin bug (Sinea diadema Fabricius), Chrysoperla carnea (Stephens), Cantharis spp …

☆ 99 Citato da 26 Articoli correlati Tutte e 8 le versioni Web of Science: 17 »

Evaluation of the functional response of selected arthropod predators on bollworm eggs in the laboratory and effect of temperature on their predation efficiency

MN Parajulee, RB Shrestha, JF Leser… - Environmental …, 2006 - academic.oup.com

… quadrimaculatus (F.), big-eyed bug, Geocoris punctipes (Say), Scymnus lady beetle, Scymnus loewii Mulsant, insidious flower bug, Orius insidiosus (Say), hooded beetle, Notoxus spp., pale damsel bug, Nabis capsiformis Germar, **leafhopper assassin bug**, Zelus renardii …

☆ 99 Citato da 61 Articoli correlati Tutte e 12 le versioni Web of Science: 37

Backyard Beneficial Insects in New Mexico

A Bennett, ML Kersten - 2019 - aces.nmsu.edu

… Figure 17B. A wheel bug sitting on goldenrod (Solidago sp.) shows the wings folded on the wider abdomen. Figure 17C. A **leafhopper assassin bug** lays in wait for prey on a plains coreopsis (Coreopsis tinctoria) flower. Figure 17D …

☆ 99 Articoli correlati »

Insect diversity in phytoremediation and bioaccumulation of Se

GS Bañuelos, JS Tebbets, JA Johnson… - International Journal …, 1999 - Taylor & Francis

… Family of insect Type of insect (genus, common name) Reduviidae Assassin bugs; Zelus renardii, predacious, beneficial **leafhopper assassin bug** Rhopalidae Plant bugs; Liorhyssus hyalinus, phytophagous, harmful hyaline grass bug Thripidae Thrips; Frankliniella occidentalis …

☆ 99 Citato da 1 Articoli correlati Tutte e 7 le versioni Web of Science: 5

Ricerche correlate

assassin bug **species** assassin bug **genus**

Presencia de Zelus renardii Kolenati (Heteroptera: Reduviidae) en Chile

T Curkovic, JE Araya, M Baena… - Boletín de la Sociedad …, 2004 - sea-entomologia.org

… 1986). En los EE.UU. se conoce vulgarmente como "**leafhopper assassin bug**", mide ca. 1,2 cm y es de color rojo a marrón amarillento (Drees y Jackman, 1999). Muchas especies del género Zelus se han identificado utilizando …

Citato da 19 Articoli correlati Tutte e 5 le versioni

Invasive true bugs (Heteroptera) established in Europe

PV Putchkov - Український ентомологічний журнал, 2013 - irbis-nbuv.gov.ua

… 10. The **leafhopper assassin bug** Zelus ren ardii (Kolenati, 1956) (Reduviidae) is a predator on various pests and useful insects in North America that was intentionally or unintentionally intro— duced to Chili, Philippines, Hawai, Samoa, Jonston island …

Citato da 5 Articoli correlati

Reduviid: An important biological control agent

K Sahayaraj, R Balasubramanian - … Rearing of Reduviid Predators for Pest …, 2016 - Springer

… Reduviid predators dwell in these important agricultural crops along with social forests. Reduviidae also are called assassin bugs, ambush bugs, or thread-legged bugs or DMK poochi, kallukatti, honeybee hunter, bee killer, caterpillar hunter, **leafhopper assassin bug**, etc …

Citato da 1 Articoli correlati

A field guide to common Texas insects

JA Jackman, BM Drees - 1998 - books.google.com

Page 1. o - | | | | BASTIAAN M. DREES, Ph.D. JOHN A. JACKMAN, Ph.D. Page 2. FIELD GUIDE TO COMMON Page 3. Gulf Publishing Company Houston, Texas Page 4. BASTIAAN M. DREES, Ph.D. JOHN A. JACKMAN, Ph.D. Page 5 …

Citato da 91 Articoli correlati Tutte e 2 le versioni

Dow AgroSciences Company Petition for Determination of Nonregulated Status of 2, 4-D and Glufosinate-Resistant DAS-81910-7 Cotton

S Abel - USDA Environmental Assessment, 2015 - aphis.usda.gov

Page 1. Dow AgroSciences Company Petition for Determination of Nonregulated Status of 2,4-D- and Glufosinate-Resistant DAS-81910-7 Cotton OECD Unique Identifier: DAS-8191Ø-7 Environmental Assessment April 2015 …

Citato da 1 Articoli correlati Tutte e 2 le versioni

A Dietary Analysis of the Microwhip Scorpion (Arachnida: Palpigradi) from Val Verde County, Texas

MP Jones - 2017 - asu-ir.tdl.org

Page 1. A DIETARY ANALYSIS OF THE MICROWHIP SCORPION (ARACHNIDA: PALPIGRADI) FROM VAL VERDE COUNTY, TEXAS A Thesis Presented to the Faculty of the College of Graduate Studies and Research Angelo State University In Partial Fulfilment of the …

Articoli correlati Tutte e 2 le versioni

MANAGE INSECTS

OY Farm - low.westernsare.org

Page 1. MANAGE INSECTS On Your Farm A Guide to Ecological Strategies Miguel A. Altieri and Clara I. Nicholls with Marlene A. Fritz Page 2. i MANAGE INSECTS ON YOUR FARM A Guide to Ecological Strategies Miguel A. Altieri and Clara I. Nicholls with Marlene A. Fritz …

Tutte e 38 le versioni

Tabla de vida del depredador Zelus renardii (KOLENATI)(Hemiptera: Heteroptera: Reduviidae) en laboratorio

CA Potin Téllez - 2008 - repositorio.uchile.cl

… 1902, se describieron de México y Hawai, respectivamente (Hart, 1986). En los EE.UU. se le conoce vulgarmente como "**leafhopper assassin bug**" (Drees y Jackman, 1999). Los adultos y las ninfas son delgados, coloridos, a menudo con áreas negruzcas, rojizas o marrones …

Citato da 1 Articoli correlati Tutte e 6 le versioni

Bayer CropScience LP Petition 17-138-01p for Determination of Nonregulated Status for Glyphosate Resistant and HPPD Inhibitor Resistant GHB811 Cotton

S Abel - 2018 - aphis.usda.gov

Page 1. Bayer CropScience LP Petition 17-138-01p for Determination of Nonregulated Status for Glyphosate Resistant and HPPD Inhibitor Resistant GHB811 Cotton OECD Unique Identifier: BCS-GH811-4 Environmental Assessment …

Articoli correlati

Artificial rearing of reduviid predators for pest management

K Sahayaraj, R Balasubramanian - 2016 - Springer

… Reduviid predators dwell in these important agricultural crops along with social forests. Reduviidae also are called assassin bugs, ambush bugs, or thread-legged bugs or DMK poochi, kallukatti, honeybee hunter, bee killer, caterpillar hunter, **leafhopper assassin bug**, etc …

Citato da 2 Articoli correlati Tutte e 3 le versioni

Segnalazioni faunistiche n. 129-137

Q di Studi, NSN della Romagna - Virology - researchgate.net

… 233 Zelus renardii fu descritto su materiale raccolto in California. La distribuzione comprende: USA, America centrale, Cile e alcune isole e atolli del Pacifico. Negli Stati Uniti, dov'è noto come "**leafhopper assassin bug**", è comune soprattutto nell'ovest e sud-ovest (HART, 1986) …

Tutte e 6 le versioni

The Flora and Fauna of Scottsdale's McDowell Sonoran Preserve

MCS ConServanCy - A Project of the McDowell Sonoran …, 2014 - mcdowellsonoran.org

Page 1. The Flora and Fauna of Scottsdale's McDowell Sonoran Preserve McDowell Sonoran Conservancy People Preserving Nature In partnership with the City of Scottsdale Page 2. The flora and fauna survey of Scottsdale's McDowell Sonoran …

Citato da 2 Articoli correlati Tutte e 2 le versioni

Good Garden Bugs: Everything You Need to Know about Beneficial Predatory Insects

M Gardiner - 2015 - books.google.com

Page 1. - - - º _\ ^*. - tº - " " " - tº º --- "Lººs - º ºr * º | - - - º lºº - | ºf lº - - - - º º' W. * … º. º | º º: - -- … º. sº º L º - * º º º º º º Wºº- TV º - º, -s; - - - N. Everything You Need - to Know About Beneficial Predatory Insects ºvary M. Gaºgº. Page 2. Page 3. Good Garden Bugs Page 4 …

Citato da 2 Articoli correlati Tutte e 2 le versioni

Crop Loss Relationships and Economic Injury Levels for *Ferrisia gilli* (Hemiptera: Pseudococcidae) Infesting Pistachio in California

DR Haviland, RH Beede… - Journal of economic …, 2015 - academic.oup.com

… In 2006 and 2007 lacewing larvae (Chrysopidae) and the **leafhopper assassin bug**, Zelus renardii Kolenati, were periodically seen associated with F. gilli , though not in sufficient density to have a major impact on mealybug density or crop loss estimates …

Citato da 1 Articoli correlati Tutte e 7 le versioni Web of Science: 1

Cotton facts

MR Chaudhry, A Guitchounts - 2003 - icac.org

Page 1. M. Rafiq Chaudhry Andrei Guitchounts International Cotton Advisory Committee CottonFacts Page 2. CottonFacts Page 3. M. Rafiq Chaudhry Andrei Guitchounts International Cotton Advisory Committee CottonFacts Technical Paper No. 25 of the …

Citato da 28 Articoli correlati Tutte e 3 le versioni
