# Supplementary material for: A biological control model to manage the vector and the infection of Xylella fastidiosa on olive trees
Source: PLoS One. 2020 Apr 30;15(4):e0232363. doi: 10.1371/journal.pone.0232363 (PMC7192417; doi:10.1371/journal.pone.0232363)
Supplement: S3 File — (PDF) [file pone.0232363.s010.pdf]

Crop Protection Compendium

Datasheet report for Zelus renardii (leafhopper assassin bug)

Top of page

Pictures

Picture	Title	Caption	Copyright

Top of page

Identity

Preferred Scientific Name

Zelus renardii Kolenati

Preferred Common Name

leafhopper assassin bug

EPPO code

ZELURE (Zelus renardii)

Top of page

Taxonomic Tree

- Domain: Eukaryota
  - Kingdom: Metazoa
    - Phylum: Arthropoda
      - Subphylum: Uniramia
        - Class: Insecta
          - Order: Hemiptera
            - Suborder: Heteroptera
              - Family: Reduviidae
                - Genus: Zelus
                  - Species: Zelus renardii

Top of page

Distribution Table

The distribution in this summary table is based on all the information available. When several references are cited, they may give conflicting information on the status. Further details may be available for individual references in the Distribution Table Details section which can be selected by going to Generate Report.

Last updated: 10 Jan 2020

Continent/Country/Region	Distribution	Last Reported	Origin	First Reported	Invasive	Reference	Notes
**Asia**							
Israel (/cpc/datasheet/108457)	Present			2018		CABI (Undated)	Original citation: van der Heyden (2018)
**Europe**							
Albania (/cpc/datasheet/108354)	Present			2016		CABI (Undated)	Original citation: van der Heyden (2017)
Greece (/cpc/datasheet/108443)	Present					CABI (Undated)	Original citation: van der Heyden (2015)
-Crete (/cpc/datasheet/108711)	Present			2014		CABI (Undated)	Original citation: van der Heyden (2015)
**North America**							
Canada (/cpc/datasheet/108388)	Present					CABI Data Mining (Undated)	
United States (/cpc/datasheet/108597)	Present					CABI (Undated a)	
-Arizona (/cpc/datasheet/108798)	Present					CABI Data Mining (Undated)	
-California (/cpc/datasheet/108799)	Present					CABI (Undated a)	
-Hawaii (/cpc/datasheet/108806)	Present					CABI (Undated a)	

Top of page

Distribution Table Details

Last updated: 10 Jan 2020

Continent/Country /Region	Distribution	Last Reported	Origin	First Reported	Invasive	Reference	Notes
**Asia**							
Israel (/cpc/datasheet /108457)	Present			2018		CABI (Undated)	Original citation: van der Heyden (2018)
**Europe**							
Albania (/cpc/datasheet /108354)	Present			2016		CABI (Undated)	Original citation: van der Heyden (2017)
Greece (/cpc/datasheet /108443)	Present					CABI (Undated)	Original citation: van der Heyden (2015)
-Crete (/cpc/datasheet /108711)	Present			2014		CABI (Undated)	Original citation: van der Heyden (2015)
**North America**							
Canada (/cpc/datasheet /108388)	Present					CABI Data Mining (Undated)	
United States (/cpc/datasheet/108597)	Present					CABI (Undated a)	
-Arizona (/cpc/datasheet /108798)	Present					CABI Data Mining (Undated)	
-California (/cpc/datasheet/108799)	Present					CABI (Undated a)	
-Hawaii (/cpc/datasheet /108806)	Present					CABI (Undated a)	

Top of page

Natural enemy of

Species	Stages attacked	Countries where known to occur	References
Aphis gossypii (/cpc/datasheet/6204)	Adults/Nymphs		
Aphytis aonidiae (/cpc/datasheet/6607)			
Aphytis vandenboschi (/cpc/datasheet/6644)			
Bemisia tabaci (/cpc/datasheet/8927)	Adults/Nymphs		
Drosophila melanogaster (/cpc/datasheet/19938)			
Glycaspis brimblecombei (/cpc/datasheet/25242)	Adults/Nymphs		
Helicoverpa armigera (/cpc/datasheet/26757)			
Heliothis virescens (/cpc/datasheet/26774)			
Heteropsylla cubana (/cpc/datasheet/27919)	Adults/Nymphs		
Pectinophora gossypiella (/cpc/datasheet/39417)			
Spodoptera exigua (/cpc/datasheet/29808)			

Top of page

References

Davranoglou LR, 2011. Zelus renardii (Kolenati, 1856), a New World reduviid discovered in Europe (Hemiptera: Reduviidae: Harpactorinae). Entomologist's Monthly Magazine, 147(1766-68):157-162. http://www.pemberleybooks.com (/cpc/abstract/20113409335)

van der Heyden, T., 2015. A recent record of Zelus renardii (Kolenati, 1856) on Crete/Greece (Hemiptera: Heteroptera: Reduviidae: Harpactorinae). (Ein aktueller Nachweis von Zelus renardii (Kolenati, 1856) auf Kreta/Griechenland (Hemiptera: Heteroptera: Reduviidae: Harpactorinae)). BVnPC, 4(52), 55-59. https://www.biodiversidadvirtual.org/taxofoto/sites/default/files/ein_aktueller_nachweis_von_zelus_renardii_kolenati_1856_auf_kreta_griechenland.pdf (https://www.biodiversidadvirtual.org/taxofoto/sites/default/files/ein_aktueller_nachweis_von_zelus_renardii_kolenati_1856_auf_kreta_griechenland.pdf)

van der Heyden, T., 2017. First records of Zelus renardii (Kolenati, 1856) (Hemiptera: Heteroptera: Reduviidae: Harpactorinae) for Albania. Arquivos Entomoloxicos, 18, 49-50. http://www.aegaweb.com/arquivos_entomoloxicos/ae18_2017_van_der_heyden_first_records_zelus_renardii_albania_hemiptera_reduviidae.pdf (http://www.aegaweb.com/arquivos_entomoloxicos/ae18_2017_van_der_heyden_first_records_zelus_renardii_albania_hemiptera_reduviidae.pdf)

van der Heyden, T., 2018. First record of Zelus renardii Kolenati (Heteroptera: Reduviidae: Harpactorinae) in Israel. Revista Chilena de Entomología, 44(4), 463-465. https://biotaxa.org/rce/article/view/43714/37633 (https://biotaxa.org/rce/article/view/43714/37633)

Distribution References

CABI Data Mining, Undated. CAB Abstracts Data Mining.,

CABI, Undated. Compendium record. Wallingford, UK: CABI

CABI, Undated a. CABI Compendium: Status as determined by CABI editor. Wallingford, UK: CABI

World Map

Analyzed by: Extent

○ Not recorded

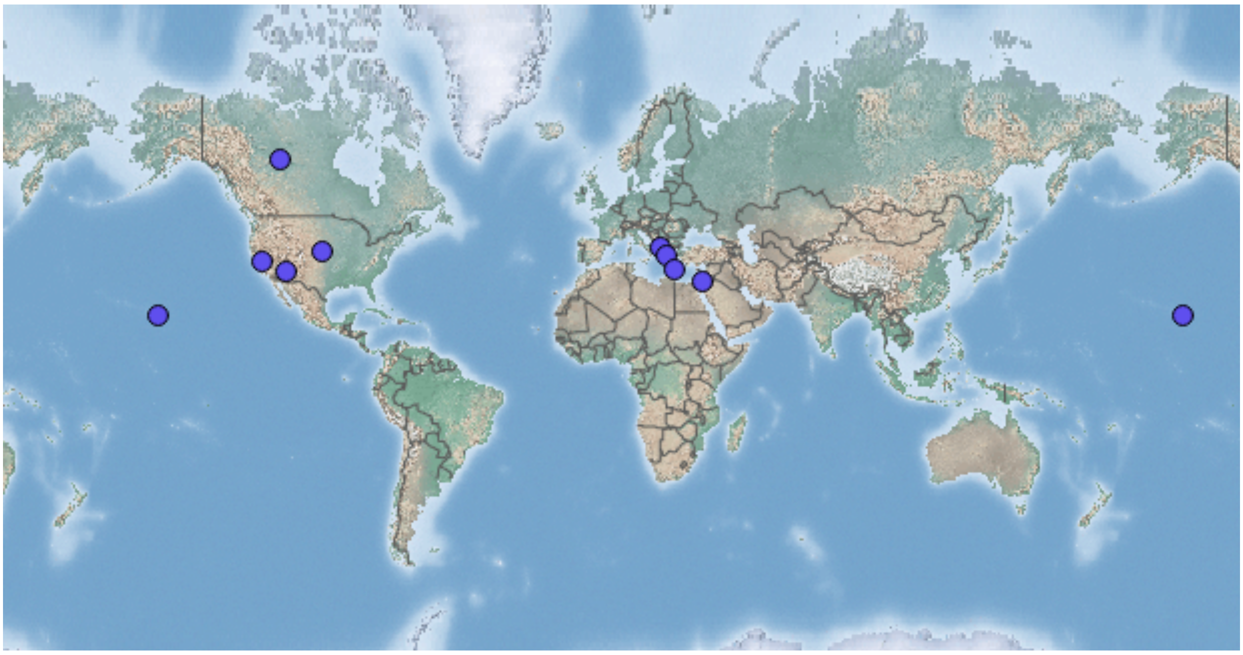

Africa

Analyzed by: Extent

○ Not recorded

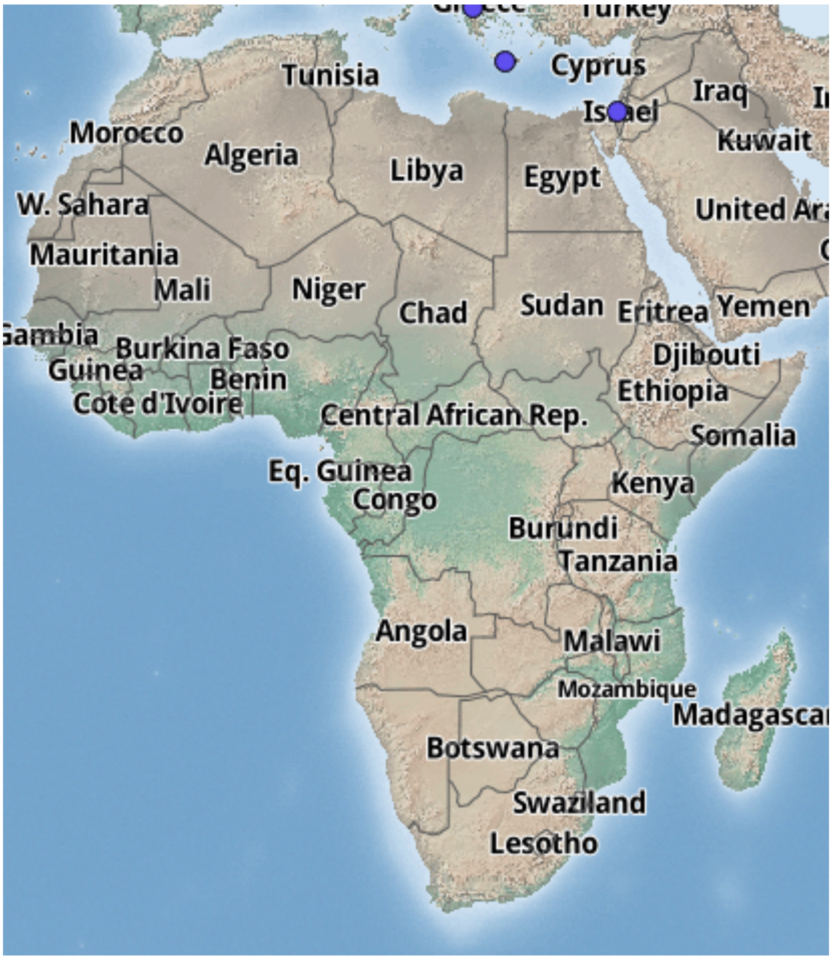

Asia

Analyzed by: Extent

○ Not recorded

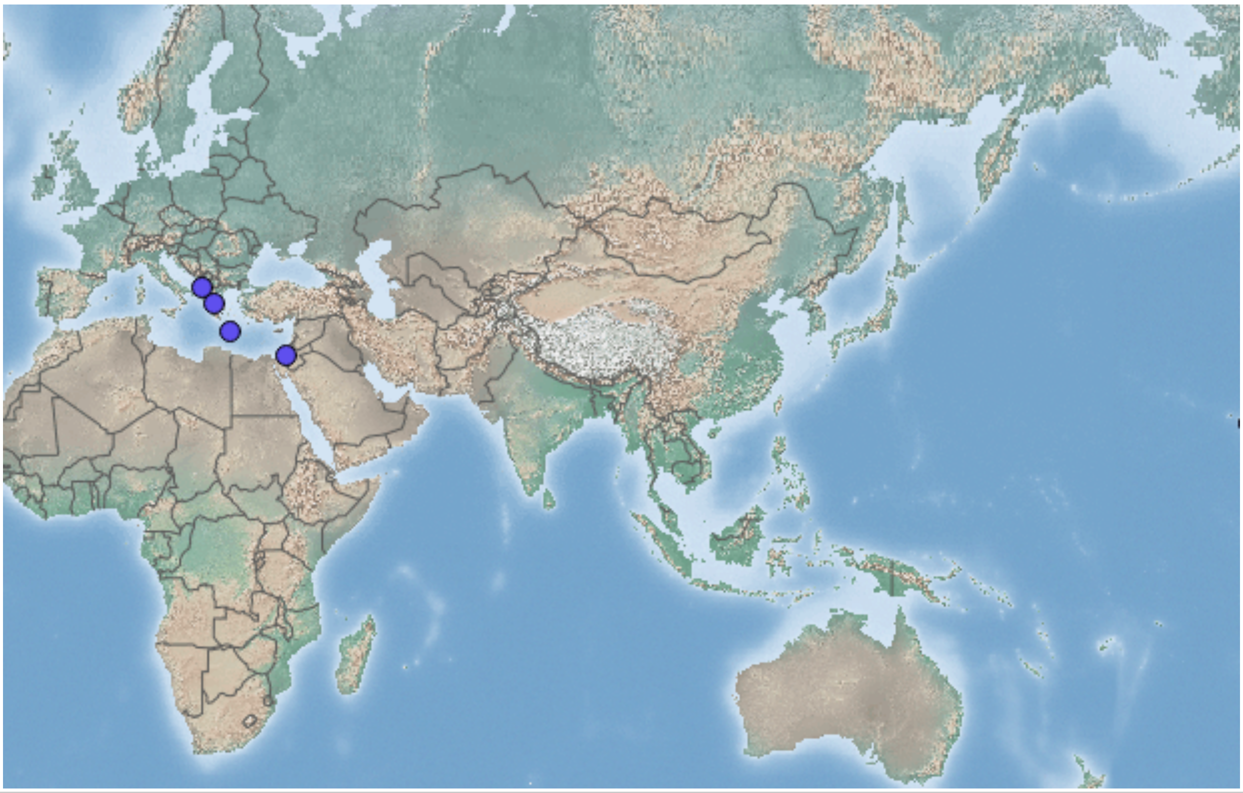

Europe

Analyzed by: Extent

Not recorded

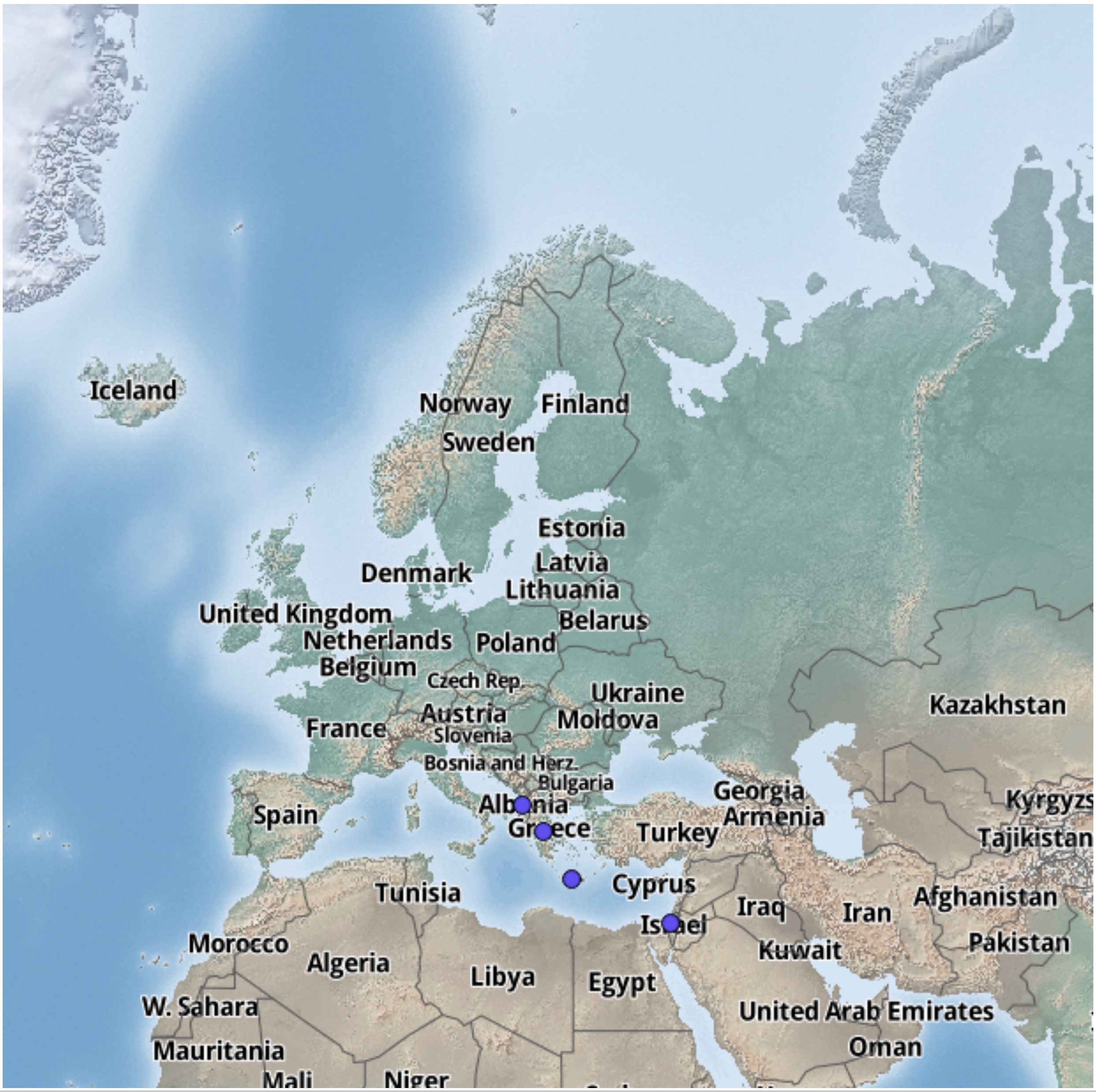

Pacific

Analyzed by: Extent

○ Not recorded

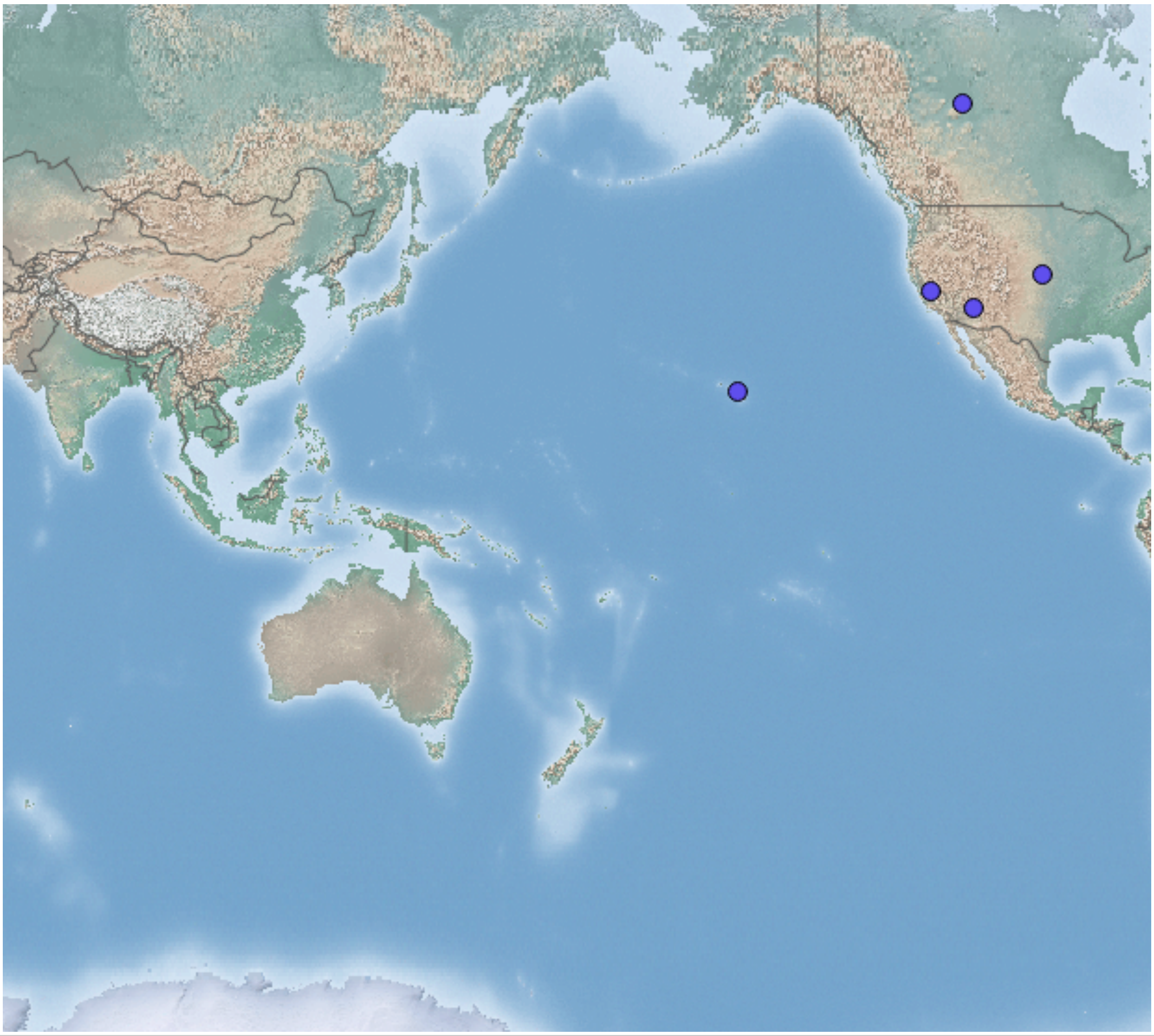

North America

Analyzed by: Extent

Not recorded

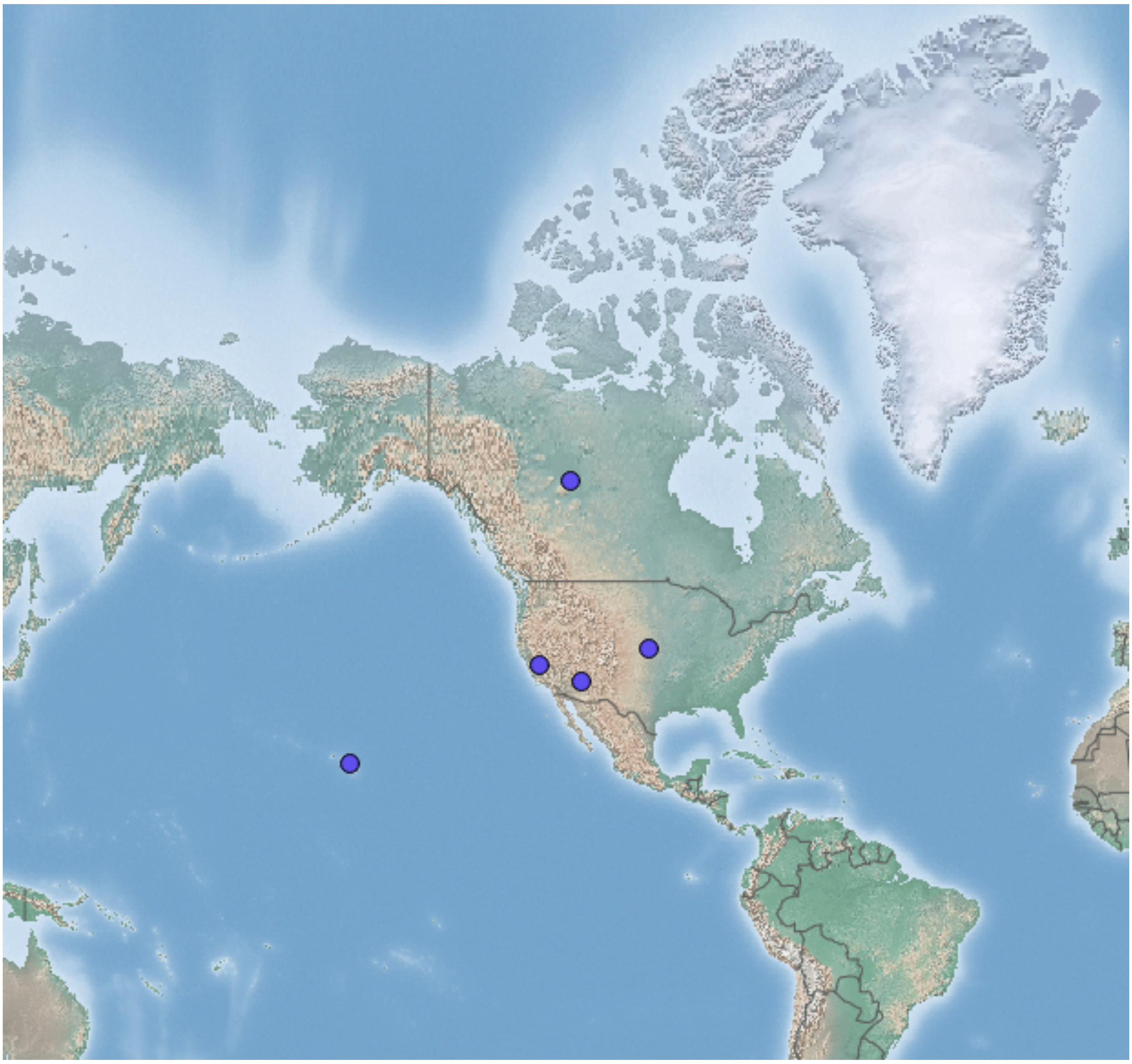

Central America

Analyzed by: Extent

Not recorded

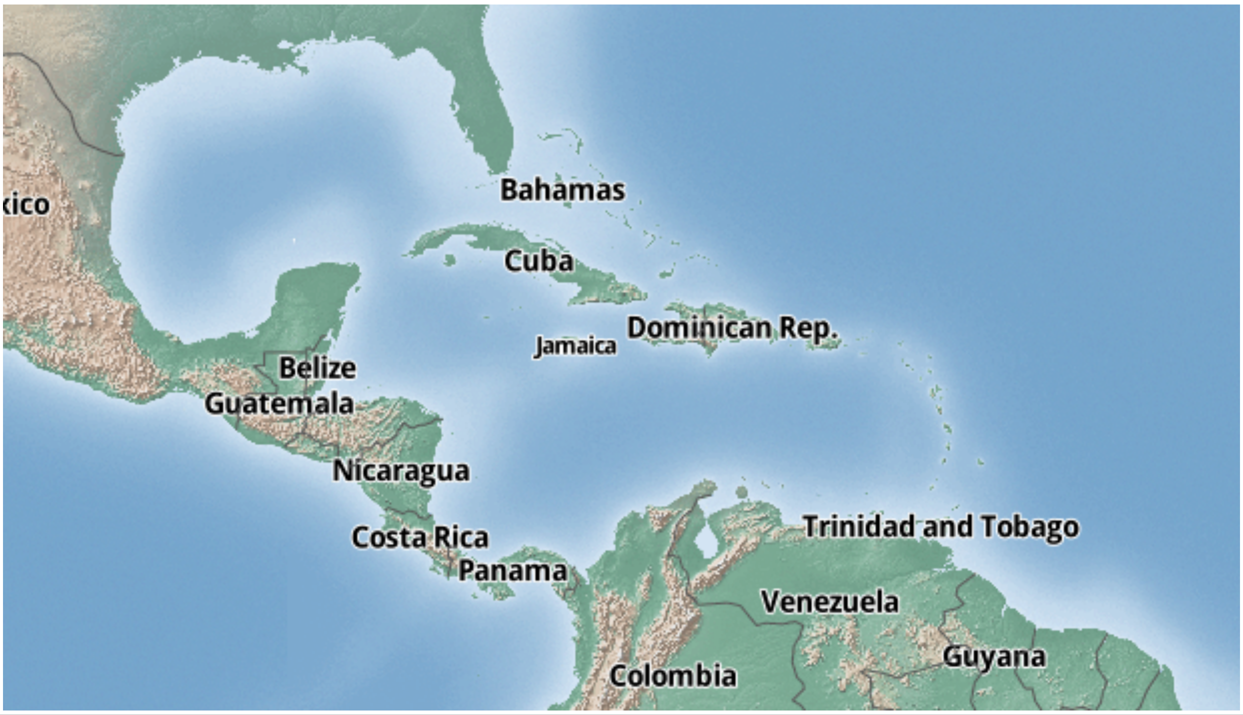

South America

Analyzed by: Extent

▢ Not recorded

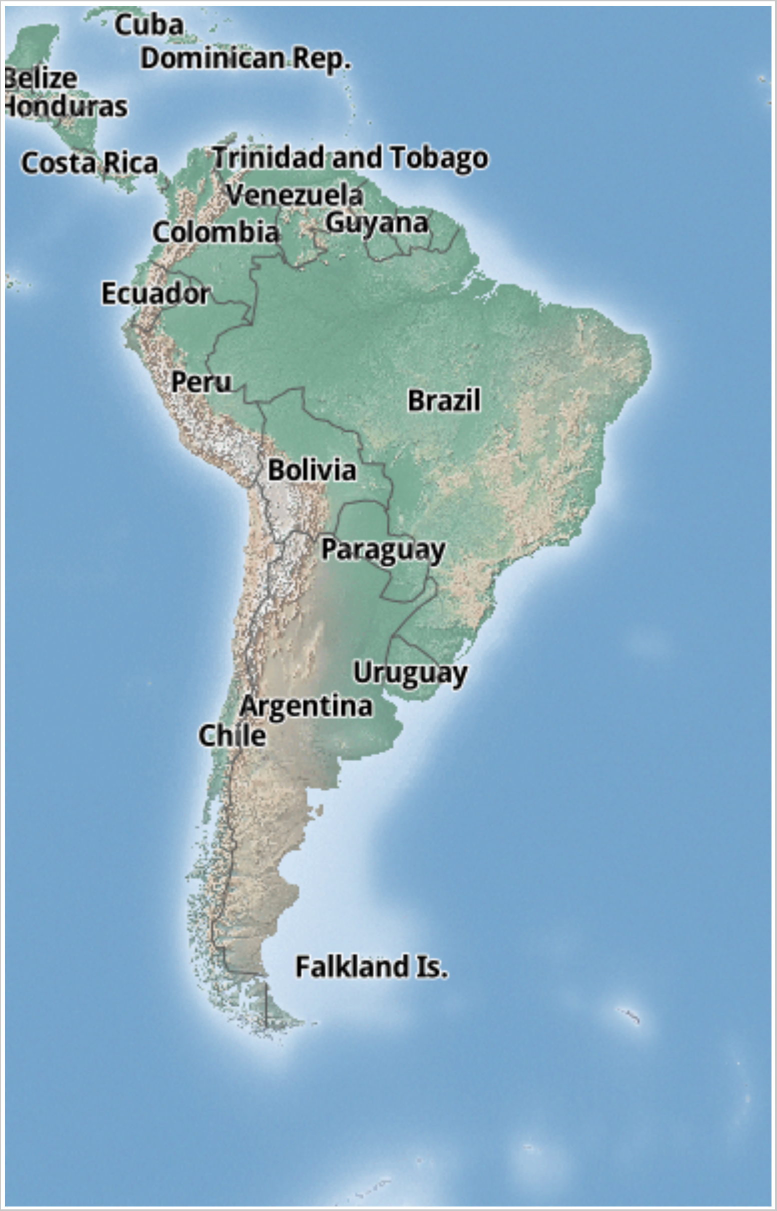

Date of report: 09 March, 2020
